# Essentials Oils from Brazilian *Eugenia* and *Syzygium* Species and Their Biological Activities

**DOI:** 10.3390/biom10081155

**Published:** 2020-08-06

**Authors:** Jamile S. da Costa, Ellen de Nazaré S. da Cruz, William N. Setzer, Joyce Kelly do R. da Silva, José Guilherme S. Maia, Pablo Luis B. Figueiredo

**Affiliations:** 1 Programa de Pós-Graduação em Ciências Farmacêuticas, Universidade Federal do Pará, Belém 66075-900, Brazil; jamile.s.costa@hotmail.com (J.S.d.C.); gmaia@ufpa.br (J.G.S.M.); 2 Programa Institucional de Bolsas de Iniciação Científica, Universidade Federal do Pará, Belém 66075-900, Brazil; ellen.cruz@icen.ufpa.br; 3 Department of Chemistry, University of Alabama in Huntsville, Huntsville, AL 35899, USA; wsetzer@chemistry.uah.edu; 4 Aromatic Plant Research Center, 230 N 1200 E, Suite 100, Lehi, UT 84043, USA; 5 Programa de Pós-Graduação em Química, Universidade Federal do Pará, Belém 66075-900, PA, Brazil; joycekellys@ufpa.br; 6 Programa de Pós-Graduação em Química, Universidade Federal do Maranhão, São Luís 64080-040, Brazil; 7 Departamento de Ciências Naturais, Universidade do Estado do Pará, Belém 66050-540, Brazil

**Keywords:** *Eugenia* spp, *Syzygium* spp, Myrtaceae, essential oil variability, mono- and sequiterpenes, biological properties

## Abstract

The *Eugenia* and *Syzygium* genera include approximately 1000 and 1800 species, respectively, and both belong to the Myrtaceae. Their species present economic and medicinal importance and pharmacological properties. Due to their chemical diversity and biological activity, we are reporting the essential oils of 48 species of these two genera, which grow in South America and found mainly in Brazil. Chemically, a total of 127 oil samples have been described and displayed a higher intraspecific and interspecific diversity for both *Eugenia* spp. and *Syzygium* spp., according to the site of collection or seasonality. The main volatile compounds were sesquiterpene hydrocarbons and oxygenated sesquiterpenes, mainly with caryophyllane and germacrane skeletons and monoterpenes of mostly the pinane type. The oils presented many biological activities, especially antimicrobial (antifungal and antibacterial), anticholinesterase, anticancer (breast, gastric, melanoma, prostate), antiprotozoal (*Leishmania* spp.), antioxidant, acaricidal, antinociceptive and anti-inflammatory. These studies can contribute to the rational and economic exploration of *Eugenia* and *Syzygium* species once they have been identified as potent natural and alternative sources to the production of new herbal medicines.

## 1. Introduction

From the latter 19th century through the second half of the 20th century, *Syzygium* species were included in the *Eugenia* genus. However, anatomical and morphological analyses provided evidence that these genera were not closely linked, and a distinction between individuals belonging to these two genera has been attributed [1]. *Eugenia* and *Syzygium* genera belong to the Myrtaceae family, included in the Myrtales order, Rosidae clade, and Malvidae subclade [2]. In recent studies, these genera have been classified into subgenera; *Eugenia* includes *Eugenia*, *Hexachlamys*, and *Pseudeugenia* subgenera [3], while the *Syzygium* genus encompasses *Syzygium*, *Acmena*, *Sequestratum*, *Perikion*, *Anetholea*, and *Wesa* subgenera [4].

*Eugenia* L. has approximately 1000 species, occurring in Central and South America, and few in the African continent [5]. They can be shrubs or small trees, twigs glabrous or pubescent when young. Leaves are opposite, petiolate; lamina gland-dotted, intramarginal vein visible. Inflorescence is axillary; solitary flowers, fascicled or rarely in triads; pubescent peduncles; pubescent bracts, persistent. Flowers are 4-merous, buds usually turbinate; rounded sepals, sparsely pubescent; petals ± orbicular, gland-dotted, and ciliate margins—stamens in multiple whorls on a broad staminal disc; ovary 2-locular, ovules several to many, radiating from a centrally-located axile placenta; style about as long as the stamens or slightly longer; stigma not dilated. Fruits are succulent berries; sepals persistent; seed 1–2, cotyledons of uniform texture, partly fused [6].

*Eugenia* species have great ecological relevance because their fruits are sources of food for birds, mammals, and reptiles [7]. Moreover, this genus stands out due to the commercial exploitation of edible fruits, wood, essential oils, and plants for ornamental purposes, and its pharmacological potential [8,9]. For example, infusions and teas of leaves, fruits, and trunk bark of *Eugenia brasiliensis* are used in Amazon folk medicine to treat stomach diseases, as an antirheumatic, anti-inflammatory, antidiarrheal, and diuretic. In contrast, decoctions of twigs of *Eugenia supraaxillaris* (syn. *Eugenia axillaris*) are used as antirheumatic and bathing by women after childbirth [10].

*Syzygium* Gaertn. has 1800 species [11], mainly found in the southern and Southeast Asia, southern China, Australia, Malaysia and New Caledonia, and some in East Africa, Madagascar, Mascarenhas Islands, southwest Pacific islands, Taiwan, and southern Japan [12]. There are only four species occurring in South America: *Syzygium aqueum* (syn. *Eugenia aquea*, *Jambosa aquea*), *S. cumini* (syn. *E. jambolana, Syzygium jambolanum*), *S. jambos (Eugenia jambos, E. monantha, Syzygium merrillii, S. monanthum)*, and *S. malaccense* [13]. 

Although *Syzygium* species are not native to the Americas, their species are well spread throughout South America in afforestation and decoration. *Syzygium cumini* and *S. malaccense* are two of the most widespread and popularly known exotic species throughout the Brazilian territory and commonly found in indigenous villages in the sub-spontaneous state amid natural vegetation [14].

*Syzygium* species can be trees, with thick, granular bark; twigs usually glabrous. Leaves are opposite, entire, penninerved, usually gland-dotted; lateral nerves united, forming a clear or faint intramarginal vein. Flowers are bisexual, in terminal or axillary corymbose cymes or panicles; calyx tube hemispherical, globose, or turbinate, tube produced above the summit of the ovary, lobes four or five, ovate to suborbicular, imbricate; petals four or five, orbicular, pellucid-glandular; stamens numerous, filaments inflexed in bud; staminal disc broad or absent; anthers globose; ovary inferior, two-celled; ovules few to several in each cell; style 1, subulate, stigma simple. The fruit is a berry, one-celled; seeds few [15]. 

*Syzygium* is one of the most common tree genera in the forest ecosystem, presenting nectariferous flowers, often in mass and typically fleshy fruits; it is used as food by birds, insects, and small and large mammals [1,12]. Its species present economic and medicinal importance [16], and pharmacological proprieties being a potential source for pharmacochemistry studies [17,18]. Traditional communities use the infusions and decoction leaves of *Syzygium cumini* and *S. aqueum* to treat diabetes, and stomach pains and dysentery, respectively [19,20].

Chemometrics analysis, such as Principal cluster analysis (PCA) and Hierarchical cluster analysis (HCA), is the most used method to group essential oils from many samples. PCA is often the first step in multivariate data analysis. The score plot is a graphical representation that provides information about relationships between samples and chemical compounds. In turn, HCA is a significant pattern recognition technique used as a preliminary evaluation of a given data set. In HCA, samples are grouped based on information that describes their relationship by similarities, such as distance and correlation. HCA is plotted in branched structures with a defined hierarchy (called dendrogram), which permits a qualitative visualization (in two-dimensional space) of grouping among samples [21]. 

**Figure 1:**
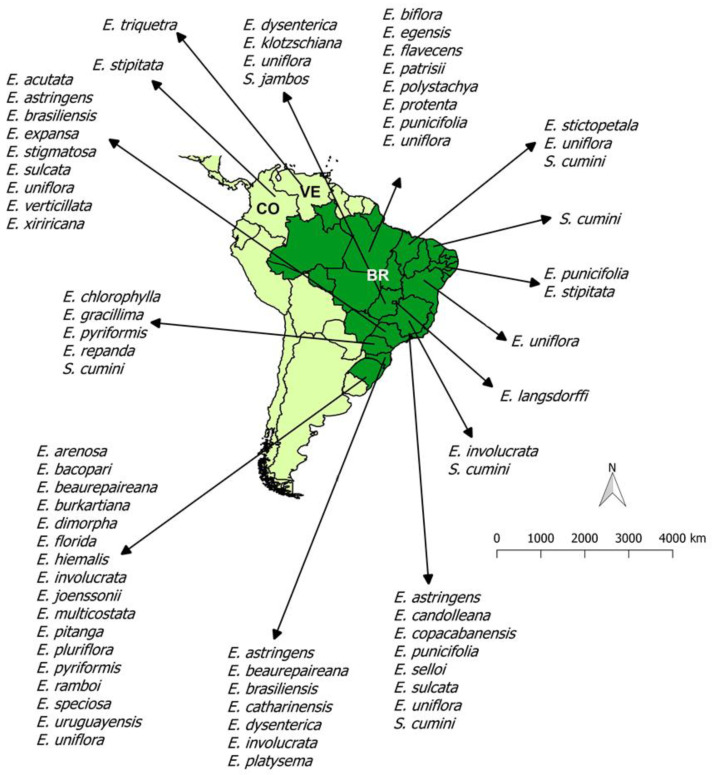
Geographical distribution in South America of *Eugenia* and *Syzygium* specimens based on their essential oil studies. This map was built by the authors using the information of the plant occurrence, available in each bibliographic reference. Abbreviation list: BR: Brazil, VE: Venezuela, CO: Colombia.

Due to the chemical and biological importance of *Eugenia* and *Syzygium* species, in this review, we report 127 chemical compositions of 48 species of these two genera growing in South America (Figure 1) and their biological activities.

## 2. Bibliographic Search Criteria

The bibliographic research was performed using the databases Google Scholar, Pubmed, Science Direct, Medline, and Scopus. The keywords applied were “*Eugenia*”, “*Syzygium*” and “essential oils”; and “volatile compounds”, and “essential oils”. Some unusual or incorrect botanical names were updated based on The Plant List” (http://www.theplantlist.org/).

## 3. Volatile Profiles 

*Eugenia* essential oils are characterized by intra- and interspecific chemical diversity, with the predominance of cyclic sesquiterpenes, follow by monoterpenes, and without phenylpropanoids as the main compound (See Appendix A and Appendix B) [22]. However, dimethylxanthoxylin (73.2 and 83.0%), an acetophenone derivative, was identified by GC–MS and NMR techniques as the major constituent in EOs of two specimens of *E. protenta* McVaugh collected in Santarém Novo, western Pará state located in the Brazilian Amazon [23].

Some essential oils of *Eugenia* species have shown great interspecific chemical variation. For example, *Eugenia protenta* EOs from Pará state (Brazil) occurring at three chemical profiles rich in the cyclic sesquiterpenes with eudesmane, elemane, and germacrane skeletons as selin-11-en-4α-ol (14.4–18.3%) and β-elemene (12.3–18.3%) (profile I); germacrene D (15.1–15.6%), bicyclogermacrene (5.8–11.8%), δ-elemene (8.5%), and β-elemene (9.2–12.8%) (profile II); or characterized by the presence of dimethylxanthoxylin (73.2–83.0%) (profile III) [23]. *E. biflora* (L.) DC. EOs from Brazil were classified in four chemical profiles as follows: caryophyllane profile, with a significant content of the sesquiterpenes (*E*)-caryophyllene (9.8–16.8%) and caryophyllene oxide (20.5–28.6%); cadinane profile, characterized by the presence of α-cadinol (14.7%); aromadendrane profile, with the predominance of the sesquiterpenes globulol (9.8%), germacrene B (7.9%) [24]; and pinane profile with β-pinene (27.8%) and α-pinene (27.3%) as main constituents [25].

Oils of *E. astringens* Cambess. (syn. *E. umbelliflora*) present two chemical profiles, three specimens collected in Rio de Janeiro and São Paulo states (Brazilian southeast) were shown to be rich in α-pinene (15.8–34.5%) and β-pinene (11.0–34.1%) [26,27,28]. The specimen from Santa Catarina (Brazil southern) was rich in viridiflorol (17.7%), followed by β- (13.2%) and α-pinene (11.2%) [29]. *E. beaurepairiana* oils from Santa Catarina state (Brazil southern) showed high amounts of cyclic sesquiterpene hydrocarbons with germacrane/caryophyllane/cadinane skeleton such as bicyclogermacrene (7.2–14.3%), germacrene D (1.6–8.6%), (*E*)-caryophyllene (6.4–8.0%), and δ-cadinene (4.9–7.2%) [30,31].

*Eugenia brasiliensis* Lam. leaf oils collected in the south and southeast Brazil displayed the occurrence of three chemical profiles. Its oils can be rich in bicyclic monoterpenes as β-pinene (10.4%) and α-pinene (10.3%) [32]; or α-pinene (18.8–35.5%), β-pinene (11.0–14.4%), followed by 1,8-cineol (9.6-28.2%) [33]; and rich in a mixture of oxygenated sesquiterpenes as spathulenol (12.7%), τ-cadinol (8.7%) and viridiflorol (7.1%) [29]. In addition, *E. brasiliensis* fruits showed different volatile profiles according to their genotypic variety. The oils extracted from the yellow variety showed as the main compound α-pinene (15.4%), myrcene (10.7%), and α–terpineol (10.2%), while oils from purple variety presented caryophyllene oxide (22.2%) and α-cadinol (10.4%) **[33]**.

A stems oil of *Eugenia chlorophylla* O.Berg from Paraná state, Brazil, was rich in oxygenated sesquiterpenes (83.4%), characterized by caryophyllene oxide (17.2%), globulol (16.5%), τ-muurolol (16.8%), α-cadinol (12.1%) and 1-*epi*-cubenol (10.9%). At the same time, it leaves oil composition at the vegetative, and flowering stages had only small quantitative differences, with globulol as the main compound at the vegetative (22.3%) and flowering (18.9%) stages. Additionally, the flowers’ oil was characterized by the predominance of sesquiterpene hydrocarbons (33.2%), such as (*E*)-caryophyllene (12.8%), α-cadinol (10.1%), caryophyllene oxide (8.9%) and τ-murolol (8.5%) [34].

Essential oils of *E. dysenterica* (Mart.) DC. leaves from central and southern regions of Brazil displayed the sesquiterpene (*E*)-caryophyllene as the main compound (14.8% and 24.0%, respectively). The minority compounds were monoterpenes with pinane (β-pinene and α-pinene), menthane (α-terpineol and limonene), caryophyllane (α-humulene and caryophyllene oxide), and cadinane skeletons (δ-cadinene, α-copaene), displaying variability in their amounts [35,36].

The oils of *E. flavescens* DC. were rich in sesquiterpenes with germacrane and bisabolane skeletons such as germacrene D (14.5%) and bicyclogermacrene (11.7%) [25], or β-bisabolene (34.7%) and (*E*)-γ-bisabolene (35.0%) [37]. Additionally, oils from *E. involucrata* DC. leaves displayed two chemical profiles rich in sesquiterpenes with germacrane skeleton. The first one rich in β-elemene (42.4%) and bicyclogermacrene (23.0%) [38], and the second rich in spathulenol (21.4%) and bicyclogermacrene (19.3%) [39]. Additionally, the oils of *E. involucrata* fruits presented a mixture of the sesquiterpenes (*E*)-caryophyllene (10.1%) and spathulenol (7.8%) as its primary constituents [40].

The moisture of plant material can influence in the EOs yield and composition. Leaves of *E. klotzschiana* O.Berg dried by three different processes (*in natura*, at room temperature, and drying by forced air circulation) showed qualitative and quantitative variations in their oil constituents. The main compounds were α-copaene (1.7-10.6%), β-bisabolene (1.9-17.4%), *trans*-α-bergamotene (0.0–10.1%), spathulenol (7.2–10.9%) and germacrene D (2.4–13.3%). Besides, there was a clear difference between the oil compositions from its leaves and flowers, which this last was rich in *trans*-α-bergamotene (29.9%) [41]. 

The EOs of leaves of *E. langsdorffii* O.Berg presented *epi*-longipinanol (13.6%), γ-eudesmol (12.3%), limonene (11.8%), and 10-*epi*-γ-eudesmol (10.6%) as the major constituents, while their fruits showed 10-*epi*-γ-eudesmol (35.7%), 1,10-di-*epi*-cubenol (15.6%) [42].

The leaf oil of *E. patrisii* Vahl was characterized by presence of cyclic sesquiterpenes with cadinane and caryophyllane skeletons including *trans*-cadin-1,4-diene (16.5%), *trans*-muurola-3,5-diene (13.3%), (*E*)-caryophyllene (11.1%) and α-cubebene (9.8%) [25]. However, its aerial parts (leaves and fine stems) had a mixture of acyclic and cyclic oxygenated sesquiterpenes as (2*E*,6*E*)-farnesol (34.5%), (2*E*,6*Z*)-farnesol (23.2%), caryophylla-4(12)-8(13)-dien-5β-ol (15.6%) as major constituents [37]. 

Different chemical oil profiles of *E. punicifolia* (Kunth) DC. were found. Specimens collected in Brazilian northeast showed linalool (44.0–61.2%), (*E*)-caryophyllene (16.2–22.7%), and α-terpineol (6.7–8.8%) as the main compounds [43]. Specimens sampled in Brazilian Amazon presented the sesquiterpene hydrocarbons (*E*)-caryophyllene (9.9%), bicyclogermacrene (8.7%), germacrene D (5.4%) and the monoterpene (*E*)-β-ocimene (5.5%) as their primary constituents [25]. Additionally, in another plant sample from Brazilian southeast predominated sesquiterpenes with cadinane/germacrane skeleton as α-cadinol (10.6%), 10-*epi*-γ-eudesmol (10.2%), paradisiol (9.0%), and 7-*epi*-α-selinene (6.8%) [28]. 

Essential oil from leaves of *E. pyriformis* Cambess. collected in southern Brazil showed two chemical profiles: The first one (1) showing sesquiterpenes with cadinane/germacrane-type skeletons as α-cadinol (14.0%), δ-cadinene (12.4%), τ-cadinol (11.9%), and bicyclogermacrene (10.2%) [30]; and the second (2) presenting the monoterpenes β-pinene (0.2–25.7%), limonene (0.2–22.0%), and 1,8-cineole (0.6–14.7%), with pinane and menthane-type skeletons, and the sesquiterpene caryophyllene oxide (3.9–21.3%) with a caryophyllane-type skeleton [44]. Additionally, flowers EO was characterized by caryophyllane/cadinane sesquiterpene-types, as (*E*)-caryophyllene (22.8%), germacrene D (15.3%), bicyclogermacrene (8.4%), and α-cadinol (7.2%) [44]. Additionally, fruits oil of *E. pyriformis* showed caryophyllene oxide (16.2%), limonene (12.4%), α-terpineol (5.4%), α-cadinol (5.4%) as the main compounds [44].

Leaf oils of *E. sulcata* Spring ex Mart. from Brazil may be characterized by two different chemical profiles: the first one (1) presented (*E*)-caryophyllene (15.0–24.6%), α-pinene (17.2–34.2%), and β-pinene (1.7–10.9%) [32,45,46], and the second (2) showed 1,8-cineole (19.0%), α-pinene (16.9%), and β-pinene (14.5%) [28]. Another sample of fine stems oil was rich in *(E*)-caryophyllene (18.8%), spathulenol (8.8%), and *trans*-calamenene (7.0%) with minor amounts of α- and β-pinene [46].

There is a significant chemical variability on the essential oils of *Eugenia uniflora* L, the most studied *Eugenia* species [22], with the occurrence of about nine chemotypes, rich in cyclic oxygenated sesquiterpenes, with a germacrane-type skeleton, as the curzerene (thermally produced in situ during the oil processing) [47,48,49,50], atractylone [51,52], selina-1,3,7(11)-trien-8-one and selina-1,3,7(11)-trien-8-one epoxide [48,49,53,54], germacrone [55,56], and in association with other cyclic sesquiterpene hydrocarbons [48,49,51,56,57,58]. 

Although various *Eugenia* species and specimens have been widely studied concerning their leaf essential oil compositions, about 26 species were sampled only once to furnish their oil compositions. These plant samples exist mainly in Brazil, and the species that included monoterpene hydrocarbons preferably were *E. dimorpha* O.Berg (α-pinene, 22.4%) [59], *E. multicostata* D.Legrand (α-pinene, 16.1% and spathulenol 10.7%) [32], *E. speciosa* Cambess. (α-pinene, 47.3% and limonene, 23.0%) [32], and *E. uruguayensis* Cambess. (α-pinene, 23.5% and β-pinene, 11.8%) [27]; and that included sesquiterpene hydrocarbons were *E. acutata* Miq. (*E*)-caryophyllene, 26.93%) [60], *E. bacopari* D.Legrand (δ-cadinene, 15.8% and aromadendrene, 12.2%) [59], *E. burkartiana* (D.Legrand) D.Legrand (bicyclogermacrene, 14.2%, germacrene D, 8.8% and (*E*)-caryophyllene, 7.8%) [59], *E. florida* DC. (*E*-caryophyllene, 14.5% and β-elemene, 1.8%) [61], *E. hiemalis* Cambess. (bicyclogermacrene, 37.7%) [62], *E. pitanga* (O.Berg) Nied. (germacrene D, 29.3% and bicyclogermacrene, 22.4%) [32], *E. platysema* O.Berg (β-selinene, 17.9%, *allo*-aromadendrene, 12.6% and 7-*epi*-α-selinene, 10.4%) [27], *E. ramboi* D.Legrand (β-elemene, 10.6%, bicyclogermacrene, 9.7% and (*E*)-caryophyllene, 6.2%) [27], *E. repanda* O.Berg ((*E*)-caryophyllene, 16.3%, α-humulene, 10.2% and bicyclogermacrene, 9.4%) [61], *E. stictopetala* Mart. ex DC. (𝛾-elemene, 17.48%, (*E*)-caryophyllene, 16.46% and bicyclogermacrene, 8.11%) [63], and *E. polystachya* rich. (germacrene D, 18.4%, ishwarane, 15.7% and 7-*epi*-α-selinene, 7.5%) [37]. Moreover, eight species were found to have oxygenated sesquiterpenes as the major constituents: *E. candolleana* DC. (1-*epi*-cubenol, 77.59%) [60]. *E. egensis* DC. (5-hydroxy-*trans*-calamenene, 35.8%) [37], *E. expansa* Spring ex Mart. (spathulenol, 12.1% and (*E*)-caryophyllene, 9.2%) [32], *E. gracillima* (globulol, 8.7%, viridiflorene, 6.9%, *epi*-globulol, 6.8%, and spathulenol, 5.9%) [59], *E. joenssonii* Kausel (5-*epi*-paradisiol, 8.4%, δ-selinene 7.9%, and β-selinene, 7.2%) [59]; *E. pluriflora* DC. ((*E*)-nerolidol, 24.6% and α-pinene, 24.0%) [27], *E. verticillata* (Vell.) Angely (valerianol, 28.1% and 10-*epi*-γ-eudesmol, 12.6%) [64], and *E. xiriricana* Mattos (spathulenol, 15.4% and β-pinene, 14.1%) [32]. In addition, three species presented other constituents such as esters, carboxylic acid, and oxygenated monoterpenes as main compounds. They were *E. catharinensis* D.Legrand (ethyl palmitate, 10.5%; *trans*-α-bergamotene, 6.5%; and β-selinene, 5.9%) [59], *E. stigmatosa* DC. (physeteric acid, 90.5%) [62], and *E. triquetra* O.Berg (from Venezuela) (linalool, 17.5%, limonene, 16.9%, α-pinene, 11.6%, and β-pinene, 8.7%) [65].

Chemical profiles of oils from *Syzygium* species growing in South America are poorly described in the literature. We found seven *Syzygium cumini* accessions (specimens) growing in Brazil. It is known that the collection site and habitat affect the chemical composition of essential oils. The oils of *S. cumini* (L.) Skeels (syn. *Eugenia cumini* and *E. jambolana*) collected in Brazil (Ceará, Maranhão, and Rio de Janeiro states) showed α-pinene (22.2–48.0%) as the main constituent [66,67,68,69], while another oil of *S. cumini* collected in Minas Gerais state, Brazil, showed α-humulene (25.2%) and (*E*)-caryophyllene (16.0%) as the predominant compounds [70].

## 4. Seasonal Variation of Oil Composition

Some studies have shown variation in the chemical composition and yield of EOs of *Eugenia* and *Syzygium* affected by seasonality. Samples of fresh leaves of *E. astringens* (syn. *Eugenia rotundifolia*), with Brazil’s occurrence, were collected every three months. The oil was mainly composed of cyclic monoterpene and sesquiterpene hydrocarbons, as α-pinene (19.7–34.4%), β-pinene (20.6–34.1%), and (*E*)-caryophyllene (3.6–11.7%). The rain precipitation data displayed a significant positive and negative correlation between the (*E*)-caryophyllene and α-pinene content, respectively [26]. 

Two oil profiles of *E. brasiliensis* were monitored according to their seasonal and circadian variations. A specimen collected in Santa Catarina state, Brazil, showed α-pinene (1.77–15.94%), β-pinene (2.98–11.24%), spathulenol (8.10–18.17%), 1-*epi*-cubenol (4.83–7.46%), and τ-cadinol (10.38–15.30%) as main volatile compounds. Spathulenol was the most abundant in the spring (16.02%) and summer (18.17%), τ-cadinol in the autumn (12.83%), and α-pinene (15.94%) in the winter [71]. The oil of the specimen collected in São Paulo state, Brazil, presented spathulenol (7.0–18.0%), *trans*-α-bergamotene (2.6–19.0%), α-thujene (4.0–11.5%), and β-selinene (2.3–8.5%) as primary constituents, with a significant content variation on the seasonality study. The monoterpenes amount was higher in July, September, and November, mainly for α-thujene (10–11%) and β-pinene (6–8%), which were detected in low proportion in the other collection months. On the other hand, the number of oxygenated sesquiterpenes increased from January (45.0%) to May (56.2%) [72]. 

The oil composition of leaves of *E. pyriformis* was monthly monitored and showed as main compounds the monoterpenes β-pinene (0.0–25.7%), limonene (0.2–22.0%), and 1,8-cineole (0.6–14.7%), followed by the sesquiterpene, caryophyllene oxide (3.9–21.3%). During the annual study, the monoterpenes were predominant in nine months, excepting April, October, and December, where the sesquiterpenes showed concentrations higher than 70%. The monoterpene hydrocarbon β-pinene was the principal constituent in January (25.0%), presented meaningful content in other months, but it was absent in October [44].

Two different leaf oil profiles of *E. uniflora* were monitored during the year seasonal changes. The oil with profiles rich in curzerene (34.0–53.1%) [73], and selin-1,3,7(11)-trien-8-one (43.0%) plus selin-1,3,7(11)-trien-8-one epoxide (20.0–29.0%) [54] have a clear seasonal influence, however these profiles did not interconvert themselves. The curzerene content showed no significant difference between the dry (42.7%) and rainy (40.8%) seasons, presenting a high level of similarity by HCA analysis [73]. On the other hand, the selin-1,3,7(11)-trien-8-one plus selin-1,3,7(11)-trien-8-one epoxide profile showed a separation into two different clusters for the oils: Cluster I included samples collected in the dry season, and it was characterized by the highest percentage of spathulenol (10.0%) and caryophyllene oxide (4.0%). Cluster II grouped samples collected in the rainy season showed selina-1,3,7(11)-trien-8-one epoxide (29%) as the principal constituent. Additionally, another two subclusters separated the rainy sampling months. The first one characterized by a high percentage of selina-1,3,7(11)-trien-8-one (46.4%), and the second that was rich in selina-1,3,7(11)-trien-8-one epoxide (37.0%) [54].

The leaf oils of *Syzygium jambos* (L.) Alston, collected in different year seasons, showed an environmental influence due to the foliar nutrients (N, Mn, Co, Fe, S, and Mg), and soil nutrients (Na, Al, S and H^+,^ and Al). The main compounds were (*E*)-caryophyllene (0–10.86%), α-humulene (0.0–7.1%), α-zingiberene (0.97–17.73%), caryophyllenyl alcohol (1.48–17.14%), caryolan-8-ol (0.0–10.75%), caryophyllene oxide (0.0–5.05%), thujopsan-2-α-ol (1.01–12.19%), and heneicosane (1.73–18.0%) [74].

## 5. Differences in Oil Composition and Extractions Methods

Differences in EO’s yields and chemical composition may be associated with the extraction technique employed and its conditions. Some studies compared the extraction methods and reported their influence in yields and chemical compositions of oils [74]. The leaf oil of *E. involucrata* collected in Rio de Janeiro (Brazil) was rich in β-elemene (41.8%), bicyclogermacrene (28.4%) and (*E*)-caryophyllene (6.7%) when extracted by CO_2_ supercritical fluid. However, the oil showed quantitative variations of β-elemene (42.4%), bicyclogermacrene (23.0%), and (*E*)-caryophyllene (13.4%) when extracted by hydrodistillation [37]. 

## 6. Biological Activities

### 6.1. Antibacterial and Antifungal Activity

In the last years, antimicrobial resistance to antibiotics has been increasing due to the adaptive evolution of bacteria and fungi. For this reason, research focused on the potential of new antimicrobial agents based on natural products has been explored, particularly the essential oils [75].

Several published works have reported the potential of *Eugenia* and *Syzygium* EOs against Gram-positive and Gram-negative bacteria. Among the Gram-positive strains, *Staphylococcus aureus*, *S. mitis*, *S. sanguinis*, *S. epidermis*, *S. saprophyticus*, *Listeria monocytogenes*, *Streptococcus equi*, *S. mutans*, *S. sobrinus*, *Bacillus cereus*, *B. subtilis*, *Enterococcus faecalis*, *Mycobacterium bovis*, *Kocuria rhizophila* were evaluated. Concerning the Gram-negative strains, *Prevotella nigrescens*, *Porphyromonas gingivalis*, *Escherichia coli*, *Pseudomonas aeruginosa*, *Enterobacter aerogenes*, *Salmonella* spp., *S. enteritidis*, *S. typhimurium*, and *Neisseria gonorrhoeae* were included.

Essential oils from *Eugenia astringens*, *E. beaurepaireana*, *E. brasiliensis*, *E. chlorophylla*, *E. stipitata*, *E. uniflora,* and *Syzygium cumini* were tested against *Staphylococcus aureus* strains and showed minimal inhibitory concentrations (MICs) ranged from 119.2 to 56,000 μg/mL [29,34,57,68,76,77].

*Eugenia uniflora* oils showed low activity when tested against *Staphylococcus epidermis* (MIC 7500 µg/mL) and *S. equi* (MIC 7500 µg/mL) [51]. The oils of *E. involucrata* displayed activity against *Streptococcus mitis* (MIC 400 µg/mL), *S. sanguinis* (MIC 400 μg/mL), *Prevotella nigrescens* (MIC 100 µg/mL), and *Porphyromonas gingivalis* (MIC 100 μg/mL) [39]. The oils of *E. astringens* (MIC 477 μg/mL), *E. beaurepaireana* (MIC 556.6 μg/mL), *E. brasiliensis* (MIC 624.9–1000 μg/mL) and *E. stipitata* (inhibition halo 11 mm, tetracycline standard 7 mm) presented antibacterial activity against *Escherichia coli* [29,68,71,77]. The oil of *E. dysenterica* showed activity against *Cryptococcus neoformans* var. *neoformans* (MIC 250 µg/mL) and *C. neoformans* var. *gattii* (MIC 250 μg/mL) [35]. Oils of *E. astringens* (MIC 470 µg/mL), *E. beaurepaireana* (MIC 270 µg/mL), *E. brasiliensis* (MIC 500–1000 µg/mL), *E. stipitata* (inhibition halo 11 mm, tetracycline standard 7 mm) and *E. uniflora* (MIC 625 µg/mL) showed activity against *Pseudomonas aeruginosa* [29,67,71,76,77].

Additionally, oils of *Eugenia uniflora* presented a moderate activity against *Listeria monocytogenes* (MIC 1040 µg/mL) [57], differently of *E. stipitata* oil (inhibition halo 12 mm, tetracycline standard that showed no inhibition) [77]. Furthermore, the oils of *Eugenia uniflora* showed activity against *Streptococcus mutans* (MIC 50 µg/mL), *S. sobrinus* (MIC 50 µg/mL), and *Kocuria rhizophila* (MIC 500 µg/mL) [34]. However, it was not active against *Enterobacter aerogenes* (MIC 3.125%) and *Salmonella typhimurium* (MIC 3.125%) [78]. With respect to antifungal activity, the oils of *E. uniflora* displayed fungicidal activity against *Paracoccidioides brasiliensis* with MICs between 62.5 and 500 μg/mL [49]; and many *Candida* species, as *Candida dubliniensis* (MIC 230 µg/mL), *C. tropicalis* (MIC 900 µg/mL), *C. albicans* (MIC 1800 µg/mL), *C. glabrata* (MIC 930 µg/mL), *C. parapsilosis* (MIC 3750 µg/mL), *C. grubii* serotype A (MIC 450 µg/mL), *C. gattii* serotype C (MIC 1800 µg/mL), *C. gattii* serotype B (MIC 220 µg/mL), *C. neoformans* serotype D (MIC 110 µg/mL), *C. lipolytica* (MIC 93.7 μg/mL), and *C. guilliermondii* (MIC 109.4 μg/mL) [51,57]. Additionally, the oils of *E. chlorophylla* showed activity against *Candida albicans* (MIC 500 µg/mL) and *C. tropicalis* (MIC 500 µg/mL) [34]. 

### 6.2. Acetylcholinesterase Inhibition 

Acetylcholinesterase (AChE) acts in the final step of the nervous impulse transmission by hydrolysis of acetylcholine (ACh), a neurotransmitter. AChE inhibitors reduce the ACh level in the brain and increase its concentration in the synapses [79]. In this sense, these inhibitors represent major compounds approved for clinical use in the symptomatic management of Alzheimer’s disease and other neurodegenerative disorders [80]. In recent years, natural compounds from essential oils have shown a high anticholinesterase potential [79]. 

There are few studies on *Eugenia* and *Syzygium* oils with this focus in South America. The essential oil of *Eugenia sucata*, collected in Rio de Janeiro, Brazil, rich in (*E*)-caryophyllene (24.6%), showed antiacetylcholinesterase activity (IC_50_ 4.66 μg/mL) by the Elman colorimetric method when compared to physostigmine (IC_50_ 0.59 μg/mL), the positive control [46]. Moreover, the oil of *E. verticillata* (syn. *E. riedeliana*) from São Paulo (Brazil), rich in valerianol (28.1%), showed an IC_50_ of 67.3 μg/mL [64]. On the other hand, the oil of *E. brasiliensis* sampled in Santa Catarina, Brazil, with a seasonal variation of α-pinene (1.77–15.94%), β-pinene (2.98–11.24%), spathulenol (8.10–18.17%), 1-*epi*-cubenol (4.83–7.46%) and τ-cadinol (10.38–15.30%), displayed low antiacetylcholinesterase activity (IC_50_ >1000 μg/mL), when compared to the control galanthamine (IC_50_ 6.93 μg/mL) [71].

### 6.3. Cytotoxic Activity

The anticancer potential of essential oils has been widely studied, aiming to mitigate the resistance development to multiple drugs and side effects associated with antitumor drugs currently used [81]. Essential oils of *E. egensis, E. flavescens, E. patrisii, E. polystachya* and *E. uniflora* showed cytotoxic activity against human cancer cell lines. The main human carcinoma cell lines evaluated were colorectal (HCT-116), gastric (AGP-01), and melanoma (SKMEL-19) [37,48].

The essential oil of *E. uniflora*, sampled in Brazil, where the selin-1,3,7(11)-trien-8-one, selin-1,3,7(11)-trien-8-one epoxide, and curzerene were the primary constituents, showed high cytotoxic activity against the colorectal (HCT-116), gastric (AGP-01) and melanoma (SKMEL-19) strains, with IC_50_ values between 8.73 to 16.26 μg/mL [48]. However, another *E. uniflora* oil chemotypes, where predominated a mixture of caryophyllene oxide, selin-1,3,7(11)-trien-8-one, selin-1,3,7(11)-trien-8-one epoxide, germacrene B, curzerene and (*E*)-caryophyllene, did not show cytotoxic activity against these cells [48]. Furthermore, oils of *E. flavescens*, *E. patrisii*, and *E. polystachya*, rich in γ-bisabolene, (2*E*,6*E*)-farnesol and germacrene D, displayed activity against colorectal carcinoma (HCT-116) with IC_50_ values of 13.9, 16.4 and 10.3 μg/mL, respectively. On the other hand, the oil of *E. egensis*, rich in 5-hydroxy-*cis*-calemenene (35.8%) did not show cytotoxic activity against this cell line (IC_50_ > 25 μg/mL) [37].

### 6.4. Antiprotozoal Activity

The protozoal or parasitic diseases induce significant morbidity and mortality, being endemic to developing countries. Thus, affordable drugs have serious side effects, high cost, and low effectiveness. Moreover, there is an increased need to expand the investigations for new drug development [82]. In this way, essential oils could be promising sources of antiprotozoal agents, opening perspectives to discover more effective drugs of vegetal origin, in the treatment of diseases caused by protozoa [83].

The essential oils of *E. uniflora* and *S. cumini* showed anti-Leishmanial activity. *Eugenia uniflora* oil from Brazil, rich in curzerene (47.3%), displayed significant anti-Leishmanial activity against promastigote (IC_50_, 3.04 µg/mL) and amastigote (IC_50_ 1.92 µg/mL) forms of *Leishmania amazonensis*. The oil was 20 times more toxic to amastigotes than to healthy macrophages. Although the anti-Leishmanial activity was not mediated by nitric oxide production, the authors have suggested that macrophage activation may be involved in the anti-Leishmanial activity of *Eugenia uniflora* essential oil, as evidenced by increases in both the phagocytic capacity and lysosomal activity [47]. Moreover, the essential oil of *Syzygium cumini* collected in Maranhão state (Brazil), rich in α-pinene (31.85%), showed anti-Leishmanial activity against promastigote form of *L. amazonensis* (IC_50_ 60 µg/mL) [67].

### 6.5. Antioxidant Aactivity

The antioxidants usually act as free radical scavengers, preventing oxidative stress, decreasing the possibility of chronic and degenerative diseases, controlling the autoxidation, and interrupting the propagation of free radicals, or by inhibiting the formation of free radicals via different mechanisms. Some aromatic plants are sources of natural antioxidants [83,84,85,86]. The antioxidant activity of *Eugenia* essential oils has been evaluated by radical scavenging DPPH, ABTS, and TLC methods, lipid peroxidation by β-carotene/linoleic acid, and the iron ion reduction power.

The oil of *E. involucrata*, obtained by supercritical fluid extraction (SFE), showed inhibition of 93.6% to the β-carotene oxidation, while its oil extracted by hydrodistillation was shown to be inactive, with only 6.4% of inhibition. The main compounds were β-elemene (41.8–42.4%), bicyclogermacrene (23.0–28.4%), and (*E*)-caryophyllene (6.7–13.4%), in both oil samples. The higher activity of the sample obtained by the extraction using SFE can be attributed to the nonvolatile compounds present in the extract [38,87]. Additionally, its fruit oil was rich in (*E*)-caryophyllene (10.1%), spathulenol (7.8%), and β-bisabolene (7.2%), displaying a significant antioxidant activity by TLC-DPPH method [40].

The oils of *E. uniflora* collected in the Brazilian Amazon with different chemical profiles showed more antioxidant activity in the DPPH method than using the β-carotene/linoleic acid system [48]. Two different samples with higher content of caryophyllene oxide, selin-1,3,7(11)-trien-8-one, selin-1,3,7(11)-trien-8-one epoxide, and curzerene (50.6%) presented an inhibition of DPPH radical of 45.1% (228.3 mgTE/g) and 42.8% (217.0 mgTE/g), respectively [47]. The leaf oils rich in curzerene (36.2-53.1%) exhibited an antioxidant capacity varying from moderate (42.6%) to high (64.2%), according to seasonality and corresponding to 186.9 mgTE/g and 400.3 mgTE/g, respectively [76]. The leaf oils rich in germacrene B (18.4%), curzerene (13.4%) and (E)-caryophyllene (9.1%) exhibited an inhibition of 40.6% (205.6 mgTE/g), and the oil samples rich in selin-1,3,7(11)-trien-8-one (32.6–43.1%) and selin-1,3,7(11)-trien-8-one epoxide (21.7-30.4%), displayed a DPPH radical scavenging of 30.3–35.3%, corresponding to 153.5–178.8 mgTE/g [48]. Another specimen of *E. uniflora*, collected in South Brazil, with an oil rich in germacrene B (21.2%) and selin-1,3,7-(11)-trien-8-one epoxide (19.3%), displayed low activity in the DPPH method (IC_50_ 833 μg/mL) and high activity to reduce ABTS radical (IC_50_ 8.1 μg/mL). The results have suggested that its antioxidant activity mechanism is based on electron transfer [57]. Additionally, the EO from fruits, composed of hexadecanoic acid (11.7%), (*E*)-β-ocimene (7.4%), and α-selinene (7.2%) showed a high antioxidant potential by TLC-DPPH method [40].

The essential oil of *E. egensis*, rich in 5-hydroxy-*cis*-calemenene (35.8%), displayed a strong DPPH radical scavenging (79.6%, 216.5 mgTE/mL, 177.6 mg.BHAE/mL), about five times less than the standards Trolox and BHA [37]. On the other hand, the *E. flavescens* oil, rich in (*E*)-γ-bisabolene (35.0%) and β-bisabolene (34.7%), showed a moderate DPPH radical scavenging (45.1%, 122.6 mgTE/mL, 100.6 mgBHAE/mL). Additionally, the *E. patrisii* oil, rich in (2*E*,6*E*)-farnesol (34.5%) and (2*E*,6*Z*)-farnesol (23.2%), presented a moderate inhibition of 40.9% (111.2 mgTE/mL, 91.3 mgBHAE/mL) [37]. The oil of *E. brasiliensis* displayed a low activity against DPPH (IC_50_ > 500 μg/mL), β-carotene/linoleic acid (1.67–14.05%), and iron reducing power (60.37–94.32 mg.AA/g) methods [71]. Similarly, the oil of *E. polystachya* showed a low activity (11.5%) against the DPPH method [37].

### 6.6. Other Biological Activities 

The oils of *E. langsdorff* from Brazil showed promise as a natural acaricide, with high fumigation potential observed for the leaf oil rich in *epi*-longipinanol (13.6%), γ-eudesmol (12.3%), limonene (11.8%), and 10-*epi*-γ-eudesmol (10.6%), and the fruit oil, where predominated 10-*epi*-γ-eudesmol (35.7%) and 1,10-di-*epi*-cubenol (15.6%). The LC_50_ (median lethal concentration) values determined for the leaf and fruit oils were 1.7 μL/L and 3.06 μL/L in air. However, in the experiment based on the residual contact, the effect of the leaf oil (LC_50_ 21.90 μL/L) was less toxic than the fruit oil (LC_50_ 12.25 μL/L) [42].

The oils of *E. stictopetala* (LC_50_ 230 μg/mL) [63] and *E. triquetra* (64.9 μg/mL) were active as larvicidal against *Aedes aegypti* larvae [65]. The pure essential oil of *E. sulcata* applied as a topical treatment on the arthropods *Oncopeltus fasciatus and Dysdercus peruvianus*, caused a mortality rate of 96.7% and 80.0%, respectively [45].

**Figure 2:**
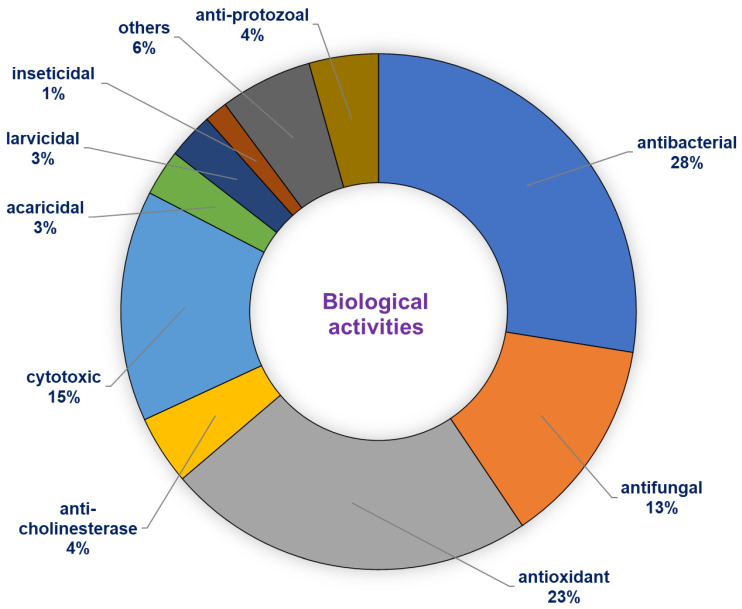
Distribution of reports on biological activity of *Eugenia* and *Syzygium* species.

The essential oils of *E. uniflora* presented antinociceptive activity (ED_50_ 218.6 mg/kg), and hypothermic [52] and hepatoprotective effects induced by acetaminophen [88] in vivo assays with mice. Moreover, the *Syzygium cumini* oil from Rio de Janeiro state, Brazil, rich in α-pinene (22.2%), (*Z*)-β-ocimene (10.2%), (*E*)-caryophyllene (9.45%) showed anti-inflammatory activity by lipopolysaccharide-induced pleurisy model, with eosinophils inhibition of 67% [66]. Additionally, *S. cumini* oil from São Luís state, Brazil, rich in α-pinene (31.85%), (*Z*)-β-ocimene (28.98%), and (*E*)-β-ocimene (11.71%), presented molluscicide potential against *Biomphalaria glabrata* (LC*_50_* 90 μg/mL) [67]. The distribution of reports on the biological activity of *Eugenia* and *Syzygium* species with occurrence in South America are represented in Figure 2.

## 7. Conclusions

The *Eugenia* and *Syzygium* species from South America have high chemical variability and several biological activities in their essential oils. Based on the present study, these variations can be attributed to different geographic occurrences or seasonal changes. Despite the medicinal use of *Eugenia* species in traditional therapy, few reports have been made about the biological activity of its essential oils (less than 5% of species in Brazil). On the other hand, the composition of oils from a large number of species (127 samples) is already known, representing 12.2% of the total species distributed in Brazil. The variations in the chemical profile of these species indicated the importance of optimizing the protocols for collecting, processing, and extracting plant material. The standardization of these essential oils can contribute to the economic and commercial exploitation of bioactive products from aromatic plants.

## Figures and Tables

**Figure A1:**
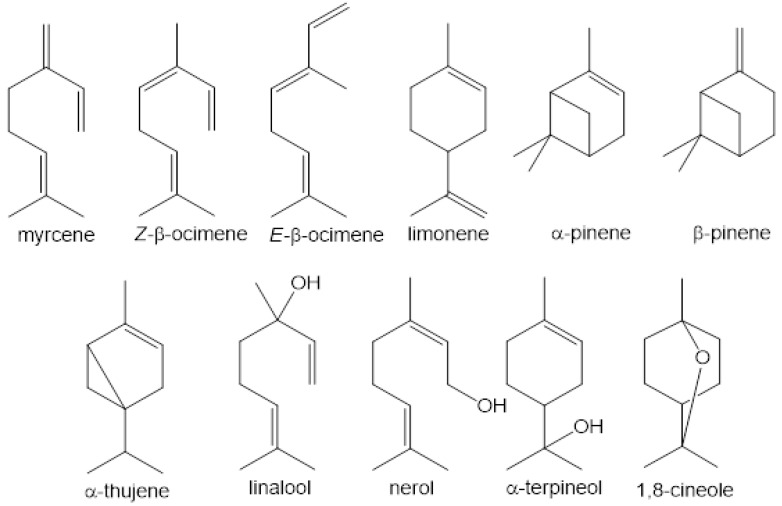
Major monoterpene constituents found in *Eugenia* and *Syzygium* essentials oils.

**Figure A2:**
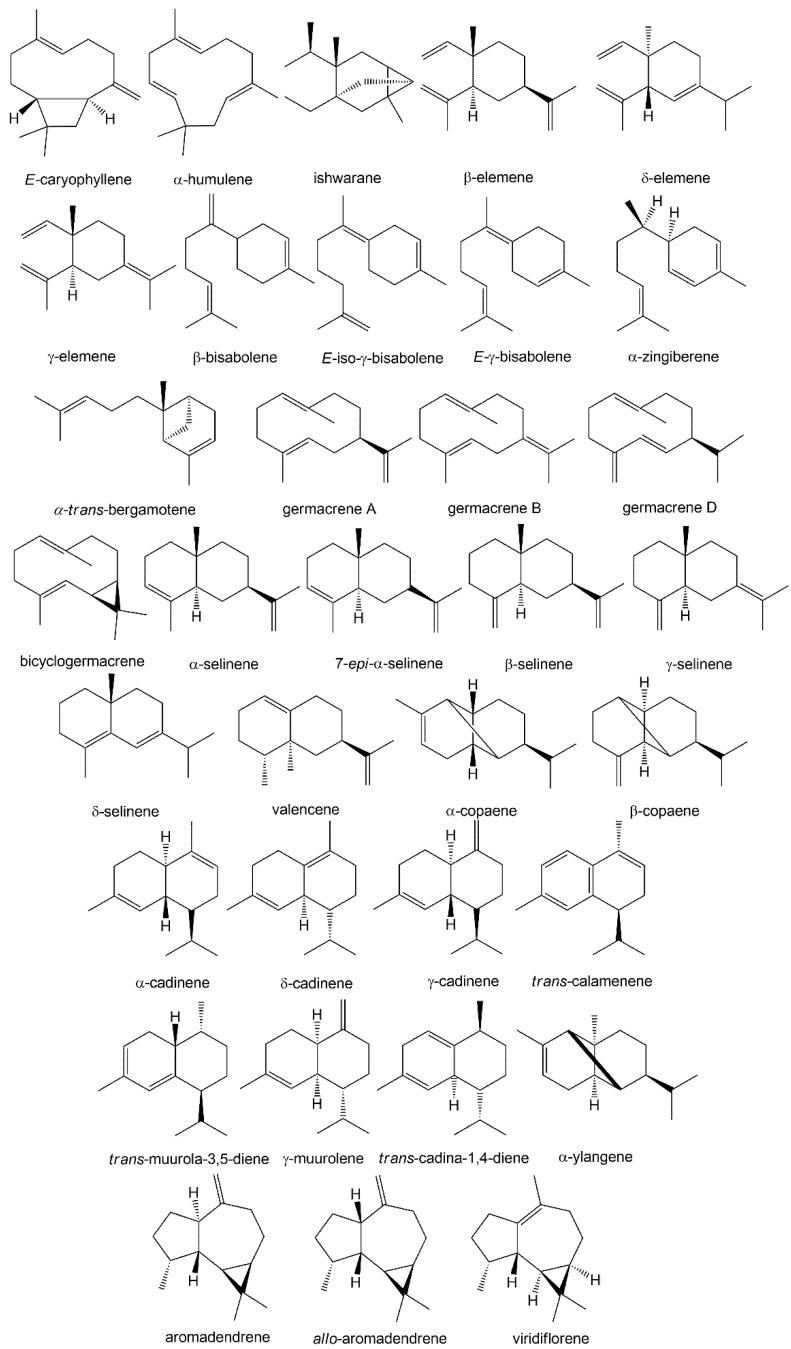
Major sesquiterpene hydrocarbons found in *Eugenia* and *Syzygium* essentials oils.

**Figure A3:**
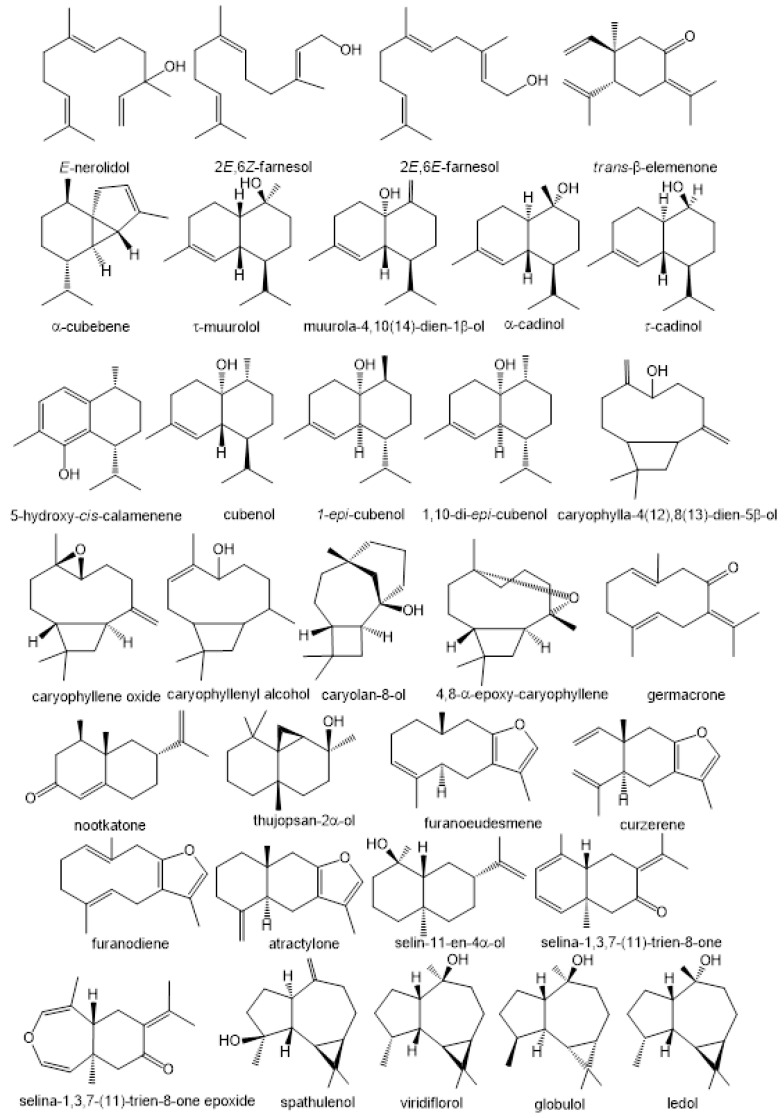

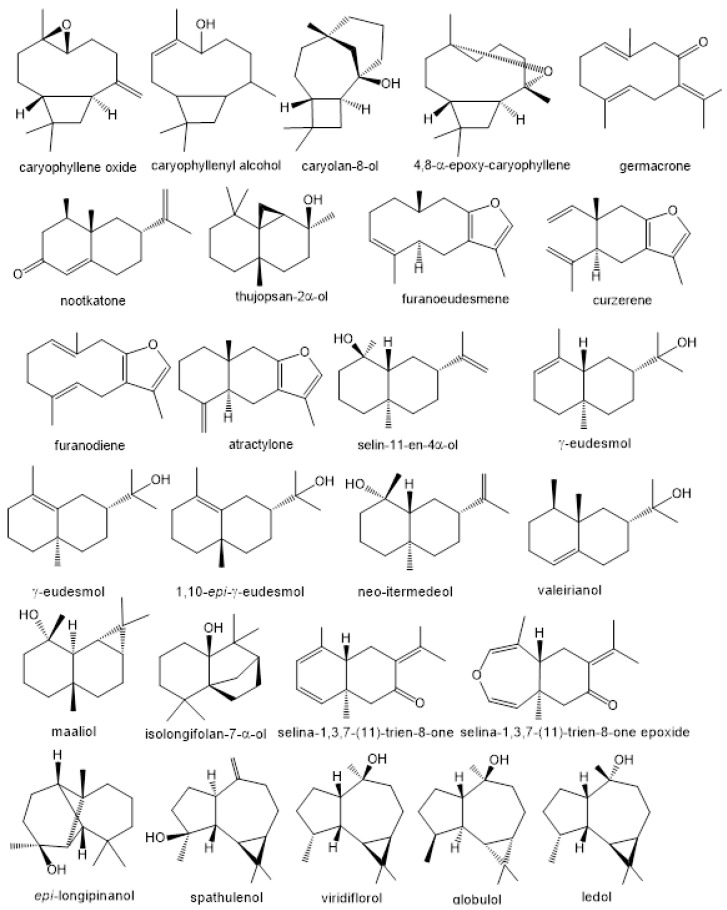
Major oxygenated sesquiterpenes found in *Eugenia* and *Syzygium* essentials oils.

**Figure A4:**
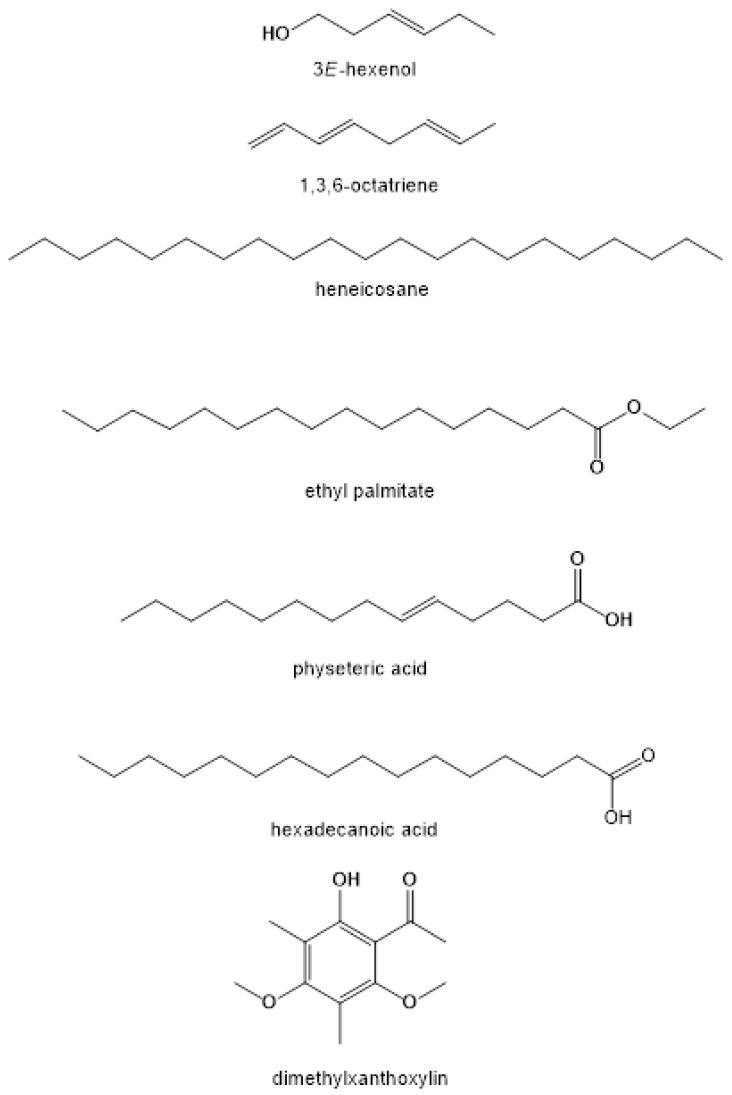
Other major constituents found in *Eugenia* and *Syzygium* essentials oils.

## Abbreviations

ABTS	2:2′-azino-bis (3-ethylbenzothiazoline-6-sulfonic acid)
ACh	Acetylcholine
AChE	Acetylcholine esterase
BHA	Butylated hydroxyanisole
BR	Brazil
CO	Colombia
DPPH	2,2-Diphenyl-1-picrylhydrazyl (radical)
ED50	Median Effective dose
EOs	Essential oils
GC–MS	Gas chromatography–mass spectrometry
HCA	Hierarchical Cluster Analysis
HD	Hydrodistillation
IC50	Median inhibitory concentration
LC50	Median lethal concentration
mg.AA/g	Milligram of ascorbic acid per gram
mg.BHAE/mL	Milligram BHA equivalente per gram
mgTE/g	Milligram trolox equivalente per gram
MIC	Minimum inhibitory concentration
MMC	Minimum microbicide concentration
NI	Unidentified compound
NMR	Nuclear magnetic resonance
PCA	Principal cluster analysis
SD	Steam distillation
SFE	Supercritical fluid extraction
SPME	Solid Phase Micro Extraction
spp.	Species (plural)
TLC	Thin-layer chromatography
VE	Venezuela

## Appendix A

**Table A1:** Species of *Eugenia* and *Syzygium* in South America: essential oil composition and biological activity.

Species	Occurrence	Essential Oil	Primary Components (>5%)	Essential Oil Bioactivity	Ref.
*E. acutata*	Valinhos, São Paulo, Brazil	Leaf (SD)	*E*-caryophyllene (26.93%), δ-cadinene (6.01%), germacrene D (5.29%)	—	[60]
*E. arenosa*	Manuel Viana, Rio Grande do Sul, Brazil	Leaf (HD)	farnesyl acetate (70.4%), aromadendrene (11.7%), globulol (7.1%)	—	[32]
*E. astringens*	Macaé, Rio de Janeiro, Brazil	Leaf (HD)	α-pinene (15.8%), 1-*epi*-cubenol (11.9%), α-eudesmol (10.6%), selin-11-en-4α-ol (9.4%)	—	[28]
*E. astringens*	Rio de Janeiro, Rio de Janeiro, Brazil	Leaf (HD) ^b^	α-pinene (19.7-34.4%), β-pinene (20.6-34.1%), *E*-caryophyllene (3.6-11.7%)	—	[26]
*E. astringens*	Juréia, São Paulo, Brazil	Leaf (HD)	α-pinene (24.7%), *E*-caryophyllene (14.6%), β-pinene (11.0%), myrcene (7.7%)	—	[27]
*E. astringens*	Santo Amaro da Imperatriz, Santa Catarina, Brazil	Leaf (HD)	viridiflorol (17.7%), β-pinene (13.2%), α-pinene (11.2%), aromadendrene (6.9%)	Antibacterial, broth dilution assay (*Staphylococcus aureus*, MIC 119.2 µg/mL; *Pseudomonas aeruginosa*, MIC 447.0 µg/mL; *Escherichia coli*, MIC 477.0 µg/mL)	[29]
*E. bacopari*	Dom Pedro de Alcantara, Rio Grande do Sul, Brazil	Leaf (HD)	δ-cadinene (15.8%), aromadendrene (12.2%), viridiflorene (7.9%), globulol (6.8%)	—	[59]
*E. beaurepaireana*	Morrinhos do Sul, Rio Grande do Sul, Brazil	Leaf (HD)	bicyclogermacrene (14.3%), germacrene D (8.6%), δ-cadinene (7.2%), *E*-caryophyllene (6.4%)	—	[30]
*E. beaurepaireana*	Santo Amaro da Imperatriz, Santa Catarina, Brazil	Leaf (HD)	*E*-caryophyllene (8.0%), bicyclogermacrene (7.2%), valencene (5.5%), δ-cadinene (4.9%), spathulenol (4.9%), viridiflorol (4.9%)	Antibacterial, broth dilution assay (*Staphylococcus aureus*, 1110 µg/mL; *Pseudomonas aeruginosa*, MIC 278.3 µg/mL; *Escherichia coli*, MIC 556.6 µg/mL)	[29]
*E. biflora*	Belém, Pará, Brazil	Leaf (HD)	caryophyllene oxide (28.6%), *E*-caryophyllene (16.8%), *τ-*-murrolol (8.4%), 1-*epi*-cubenol (6.4%), α-cadinol (6.2%)	—	[24]
*E. biflora*	Belém, Pará, Brazil	Leaf (HD)	caryophyllene oxide (20.5%), *E*-caryophyllene (11.4%), *epi*-α-murrolol (7.6%), 1-*epi*-cubenol (6.6%), α-cadinol (6.3%)	—	[24]
*E. biflora*	Belém, Pará, Brazil	Leaf (HD)	*E*-caryophyllene (9.8%), globulol (9,8%), germacrene B (7.9%), α-cadinol (7.5%)	—	[24]
*E. biflora*	Belém, Pará, Brazil	Leaf (HD)	α-cadinol (14.7%), *τ*-murrolol (8.3%), caryophyllene oxide (7.5%), copaborneol (5.6%)	—	[24]
*E. biflora*	Maracanã, Pará, Brazil	Leaf (HD)	*E*-caryophyllene (15.4%), germacrene D (6.6%), α-copaene (6.0%), bicyclogermacrene (5.8%)	—	[25]
*E. biflora*	Maracanã, Pará, Brazil	Leaf (HD)	β-pinene (27.8%), α-pinene (27.3%), limonene (6.7%)	—	[25]
*E. brasiliensis*	Blumenau, Santa Catarina, Brazil	Leaf (HD)	β-pinene (10.4%), α-pinene (10.3%), spathulenol (7.7%), τ-cadinol (7.1%)	—	[32]
*E. brasiliensis*	Florianópolis, Santa Catarina, Brazil	Leaf (HD) ^b^	α-pinene (1.77-15.94%), β-pinene (2.98-11.24%), spathulenol (8.10-18.17%), 1-*epi*-cubenol (4.83-7.46%), τ-cadinol (10.38-15.30%)	Antibacterial, broth dilution assay (*Staphylococcus saprophyticus*, MIC 500-1000 µg/mL; *Escherichia coli*, MIC 1000 µg/mL; *Pseudomonas aeruginosa*, MIC 500-1000 µg/mL) Antioxidant (DPPH IC_50_ > 500 μg/mL; β-carotene/linoleic acid, 1.67-14.05%; iron reducing power, 60.37-94.32 mg AA/g) Enzyme inhibitory (acetylcholinesterase, IC_50_ >1000 μg/mL)	[71]
*E. brasiliensis*	Moji-Guaçu, São Paulo, Brazil	Leaf Purple Variety (HD)	α-pinene (18.8%), β-pinene (11.0%), 1,8-cineol (9.6%), limonene (8.6%), τ-cadinol (6.8%)	—	[33]
*E. brasiliensis*	Moji-Guaçu, São Paulo, Brazil	Leaf Yellow Variety (HD)	α-pinene (33.5%), 1,8-cineol (28.2%), β-pinene (14.4%), myrcene (5.5%)	—	[33]
*E. brasiliensis*	Moji-Guaçu, São Paulo, Brazil	Fruit Yellow Variety (HD)	α-pinene (15.4%), myrcene (10.7%), α–terpineol (10.2%), β-pinene (9.3%)	—	[33]
*E. brasiliensis*	Santo Amaro da Imperatriz, Santa Catarina, Brazil	Leaf (HD)	spathulenol (12.7%), τ-cadinol (8.7%), viridiflorol (7.1%), 1-*epi*-cubenol (6.3%), α-cadinol (6.6%)	Antibacterial, broth dilution assay (*Staphylococcus aureus*, MIC 156.2 µg/mL; *Pseudomonas aeruginosa*, MIC 624.9 µg/mL; *Escherichia coli*, MIC 624.9 µg/mL)	[29]
*E. brasiliensis*	São Paulo, São Paulo, Brazil	Leaf (HD) ^b^	spathulenol (7.0-18.0%), *trans*−α−bergamotene (2.6-19.0%), α−thujene (4.0-11.5%), β−selinene (2.3-8.5%)	—	[72]
*E. brasiliensis*	Moji-Guaçu, São Paulo, Brazil	Fruit Purple Variety (HD)	caryophyllene oxide (22.2%), α-cadinol (10.4%), τ-cadinol (9.9%), β-bisabolene (9.6%)	—	[33]
*E. burkartiana*	Parque do Turvo, Rio Grande do Sul, Brazil	Leaf (HD)	bicyclogermacrene (14.2%), germacrene D (8.8%), *E*-caryophyllene (7.8%), 3*E*-hexenol (6.7%)	—	[59]
*E. candolleana*	Rio de Janeiro, Rio de Janeiro, Brazil	Leaf (SD)	1-*epi*-cubenol (77.59%), δ-elemene (13.87%), muurola-4,10(14)-dien-1β-ol (8.68%), globulol (5.52%), α-cadinol (5.26%)	—	[60]
*E. catharinensis*	Blumenau, Santa Catarina, Brazil	Leaf (HD)	ethyl palmitate (10.5%), trans-α-bergamotene (6.5%), β-selinene (5.9%)	—	[59]
*E. chlorophylla*	Curitiba, Paraná, Brazil	Fine Stem (HD)	caryophyllene oxide (17.2%), τ-muurolol (16.8%), globulol (16.5%), α-cadinol (12.1%), 1-*epi*-cubenol (10.9%)	Antimicrobial, agar diffusion assay and broth dilution assay (*Kocuria rhizophila*, MIC 500 µg/mL; *Staphylococcus aureus*, MIC 500 µg/mL; *Streptococcus mutans*, MIC 50.0 µg/mL; *Streptococcus sobrinus*, MIC 50.0 µg /mL; *Candida tropicalis*, MIC 50.0 µg/mL)	[34]
*E. chlorophylla*	Curitiba, Paraná, Brazil.	Leaf (HD)	globulol (22.5%), 1-10-di-*epi*-cubenol, (9.8%), τ-muurolol (8.1%), caryophyllene oxide (6.4%)	Antimicrobial, agar diffusion assay and broth dilution assay (*Kocuria rhizophila*, MIC 500, 50.0 µg /mL; *Staphylococcus aureus*, MIC 500, 50.0 µg/mL; *Streptococcus mutans*, MIC 50.0 µg/mL; *Streptococcus sobrinus*, MIC 50.0 µg/mL; *Candida albicans*, MIC 50.0 µg/mL; *Candida tropicalis*, MIC 50.0 µg/mL)	[34]
*E. chlorophylla*	Curitiba, Paraná, Brazil	Leaf at flowering stage (HD)	globulol (18.9%), α-cadinol (9.4%), 1-*epi*-cubenol (8.1%), *E*-caryophyllene (8.1%), caryophyllene oxide (5.8%)	Antimicrobial, agar diffusion assay and broth dilution assay (*Kocuria rhizophila*, MIC 500 µg/mL; *Staphylococcus aureus*, MIC 500 µg/mL; *Streptococcus mutans*, MIC 50.0 µg/mL; *Streptococcus sobrinus*, MIC 50.0 µg/mL; *Candida albicans*, MIC 50.0 µg/mL; *Candida tropicalis*, 50.0 µg/mL)	[34]
*E. chlorophylla*	Curitiba, Paraná, Brazil	Flower (HD)	*E*-caryophyllene (12.8%), α-cadinol (10.1%), caryophyllene oxide (8.9%), τ-muurolol (8.5%)	Antimicrobial, agar diffusion assay and broth dilution assay (*Kocuria rhizophila*, MIC 500 µg/mL; *Staphylococcus aureus*, MIC 500 µg/mL; *Streptococcus mutans*, MIC 50.0 µg/mL; *Streptococcus sobrinus*, MIC 50.0 µg/mL; *Enterococcus faecalis*, MIC 1000 µg/mL)	[34]
*E. copacabanensis*	Rio de Janeiro, Rio de Janeiro, Brazil	Leaf (SD)	1,10-di-*epi*-cubenol (14.24%), caryophyllene oxide (6.75%), *epi*-α-cadinol (5.03%), γ-cadinene (4.17%)	—	[60]
*E. copacabanensis*	Rio de Janeiro, Rio de Janeiro, Brazil	Leaf (HD)	β-pinene (50.4%), α-pinene (20.2%), *E*-caryophyllene (10.3%)	—	[89]
*E. dimorpha*	Morro Santana, Rio Grande do Sul, Brazil	Leaf (HD)	α-pinene (22.4%), α-humulene (12.9%), 1,8-cineole (9.9%)	—	[59]
*E. dysenterica*	Catalão, Goiás, Brazil	Leaf (HD)	*E*-caryophyllene (14.8%), α-humulene (11.0%), α-terpineol (6.1%), limonene (5.5%), δ-cadinene (5.8%), caryophyllene oxide (5.4%)	Antibacterial, broth dilution assay (*Cryptococcus neoformans* var *neoformans*, MIC 250 µg/mL; *Cryptococcus neoformans* var *gattii*, MIC 250 μg/mL)	[35]
*E. dysenterica*	Senador Canedo Santa Catarina, Brazil	Leaf (HD)	*E*-caryophyllene (18.0%), β-pinene (9.3%), α-pinene (9.0%), limonene (7.8%), *Z*-β-ocimene (5.9%)	—	[36]
*E. dysenterica*	Campo Alegre de Goiás, Goiás, Brazil	Leaf (HD)	*E*-caryophyllene (24.0%), δ-cadinene (13.0%), α-copaene (9.6%), α-pinene (6.4%), caryophyllene oxide (4.5%)	—	[36]
*E. egensis*	Marabá, Pará, Brazil	Aerial parts (HD)	5-hydroxy-*Z*-calamenene (35.8%), *E*-caryophyllene (8.9%), *trans*-muurola-3,5-diene (5.9%), *trans*-calamenene (6.1%), *trans*-cadin-1,4-diene (6.3%), ledol (5.0%)	Antioxidant, DPPH assay (79.6%, 216.5 mgTE/mL, 177.6 mgBHAE/mL) Cytotoxic (HCT-116 human colorectal carcinoma, IC_50_ > 25 μg/mL; MRC5 human fibroblast IC_50_ > 25 μg/mL; AGP-01 human gastric adenocarcinoma, IC_50_ > 25 μg/mL; SKMEL-19 human melanoma, IC_50_ > 25 μg/mL)	[37]
*E. expansa*	Peruíbe, São Paulo, Brazil	Leaf (HD)	spathulenol (12.1%), *E*-caryophyllene (9.2%), caryophyllene oxide (8.7%), α-copaene (5.0%)	—	[32]
*E. flavescens*	Maracanã, Pará, Brazil	Leaf (HD)	germacrene D (14.5%), bicyclogermacrene (11.7%), δ-cadinene (5.7%)	—	[25]
*E. flavescens*	Parauapebas, Pará, Brazil	Aerial parts (HD)	*E*-γ-bisabolene (35.0%), β-bisabolene (34.7%), *E*-*iso*-γ-bisabolene (5.1%)	Antioxidant, DPPH assay (45.1%, 122.6 mgTE/mL, 100.6 mgBHAE/mL) Cytotoxic (HCT-116 human colorectal carcinoma, IC_50_ 13.99 μg/mL; MRC5 human fibroblast IC_50_ 14.0 μg/mL; AGP-01 human gastric adenocarcinoma, IC_50_ > 25 μg/mL; SKMEL-19 human melanoma, IC_50_ >25 μg/mL)	[37]
*E. florida*	Derrubadas, Rio Grande do Sul, Brazil	Leaf (HD)	*E*-caryophyllene (14.5%), β-elemene (11.8%), α-copaene (7.9%), α-cadinol (5.8%)	—	[61]
*E. gracillima*	Porto Rico, Paraná, Brazil	Leaf (HD)	globulol (8.7%), viridiflorene (6.9%), *epi*-globulol (6.8%), spathulenol (5.9%)	—	[61]
*E. hiemalis*	Morro Santana, Rio Grande do Sul, Brazil	Leaf (HD)	bicyclogermacrene (37.7%), *E*-caryophyllene (7.4%), germacrene D (7.0%), globulol (5.8%)	—	[62]
*E. involucrata*	Urupema, Santa Catarina, Brazil	Leaf (SFE)	β-elemene (41.8%), bicyclogermacrene (28.4%), *E*-caryophyllene (6.7%), germacrene D (7.4%)	Antioxidant (β-carotene/ linoleic acid assay, at 1 mg/mL, 93.8%)	[38]
*E. involucrata*	Urupema, Santa Catarina, Brazil	Leaf (HD)	β-elemene (42.4%), bicyclogermacrene (23.0%), *E*-caryophyllene (13.4%)	Antioxidant (β-carotene/ linoleic acid assay, at 1 mg/mL, 6.41%)	[38]
*E. involucrata*	Uberlândia, Minas Gerais, Brazil	Leaf (HD)	spathulenol (21.4%), bicyclogermacrene (19.3%), *E*-caryophyllene (8.6%), caryophyllene oxide (7.7%)	Antibacterial, broth dilution assay (*Streptococcus mitis*, MIC 400 µg/mL; *S. sanguinis*, MIC 400 μg/mL; *Prevotella nigrescens*, MIC 100 µg/mL; *Porphyromonas gingivalis*, MIC 100 μg/mL)	[39]
*E. involucrata*	Pelotas, Rio Grande do Sul, Brazil,	Fruit (HD)	*E*-caryophyllene (10.1%), spathulenol (7.8%), β-bisabolene (7.2%), γ-eudesmol (6.0%)	Antioxidant, DPPH assay (TLC method)	[40]
*E. joenssonii*	Morro Santana, Rio Grande do Sul, Brazil	Leaf (HD)	5-*epi*-paradisiol (8.4%), δ-selinene (7.9%), β-selinene (7.2%), neointermedeol (6.3%), α-cadinol (5.3%)	—	[59]
*E. klotzschiana*	Portelândia, Goiás, Brazil	Leaf (HD)	α-copaene (10.6%), α-humulene (5.5%), spathulenol (8.7%), caryophyllene oxide (7.4%), *τ*-muurolol (5.4%) α-cadinol (6.2%)	—	[41]
*E. klotzschiana*	Portelândia, Goiás, Brazil	Leaf (HD)	β-bisabolene (14.0%), spathulenol (10.9%), caryophyllene oxide (6.3%), bicyclogermacrene (5.4%)	—	[41]
*E. klotzschiana*	Portelândia, Goiás, Brazil	Leaf (HD)	β-bisabolene (17.4%), germacrene D (13.3%), α-humulene (10.2%), α-*trans*-bergamotene (10.1%), spathulenol (7.2%)	—	[41]
*E. klotzschiana*	Portelândia, Goiás, Brazil	Flower (HD)	α-*trans*-bergamotene (29.9%), germacrene D (12.1%), β-bisabolene (10.2%)	—	[41]
*E. langsdorffi*	Brasília, Distrito Federal, Brazil	Leaf (HD)	*epi*-longipinanol (13.6%), γ-eudesmol (12.3%), limonene (11.8%), 10-*epi*-γ-eudesmol (10.6%), maaliol (6.2%)	Acaricidal (*Tetranychus urticae*, Fumigation LC_50_ 1.79 µL/L; residual contact LC_50_ 21.90 µL/L)	[42]
*E. langsdorffi*	Brasília, Distrito Federal, Brazil	Fruit (HD)	10-*epi*-γ-eudesmol (35.7%), 1,10-di-*epi*-cubenol (15.6%), caryophyllene oxide (7.5%), *epi*-longipinanol (7.3%), isolongifolan-7-α-ol (7.1%)	Acaricidal (*Tetranychus urticae*, Fumigation LC_50_ 3.06 µL/L; residual contact LC_50_ 12.25 µL/L)	[42]
*E. multicostata*	Dom Pedro de Alcântara, Rio Grande do Sul, Brazil	Leaf (HD)	α-pinene (16.1%), spathulenol (10.7%), *epi*-globuol (7.8%), β-pinene (7.3%)	—	[32]
*E. patrisii*	Maracanã, Pará, Brazil	Leaf (HD)	*trans*-cadin-1,4-diene (16.5%), *trans*-muurola-3,5-diene (13.3%), *E*-caryophyllene (11.1%), α-cubebene (9.8%)	—	[25]
*E. patrisii*	São Geraldo do Araguaia, Pará, Brazil	Aerial parts (HD)	2*E*,6*E*-Farnesol (34.5%), 2*E*,6*Z*-Farnesol (23.2%), caryophylla-4(12)-8(13)-dien-5β-ol (15.6%)	Antioxidant, DPPH assay (40.9%, 111.2 mgTE/mL, 91.3 mgBHAE/mL) Cytotoxic (HCT-116 human colorectal carcinoma, IC_50_ 16.4 μg/mL; MRC5 human Fibroblast IC_50_ 18.1 μg/mL; AGP-01 human gastric adenocarcinoma, IC_50_ > 25 μg/mL; SKMEL-19 human melanoma, IC_50_ > 25 μg/mL)	[37]
*E. pitanga*	Manuel Viana, Rio Grande do Sul, Brazil	Leaf (HD)	germacrene D (29.3%), bicyclogermacrene (22.4%), *E*-β-ocimene (10.5%), *E*-caryophyllene (5.1%)	—	[32]
*E. platysema*	Blumenau, Santa Catarina, Brazil	Leaf (HD)	β-selinene (17.9%), *allo*-aromadendrene (12.6%), 7-*epi*-α-selinene (10.4%)	—	[27]
*E. pluriflora*	Aparados da Serra, Rio Grande do Sul, Brazil	Leaf (HD)	*E*-nerolidol (24.6%), α-pinene (24.0%), 1,8-cineole (12.7%), α-terpineol (5.8%)	—	[27]
*E. polystachya*	Parauapebas, Pará, Brazil	Aerial parts (HD)	germacrene D (18.4%), ishwarane (15.7%), 7-*epi*-α-selinene (7.5%), bicyclogermacrene (5.1%), α-ylangene (5.0%)	Antioxidant, DPPH assay (11.5%) Cytotoxic (HCT-116 human colorectal carcinoma, IC_50_ 10.3 μg/mL; MRC5 human fibroblast IC_50_ > 25 μg/mL; AGP-01 human gastric adenocarcinoma, IC_50_ > 25 μg/mL; SKMEL-19 human melanoma, IC_50_ > 25 μg/mL)	[37]
*E. protenta*	Maracanã, Pará, Brazil	Leaf (HD)	germacrene D (15.1%), β-elemene (12.8%), δ-cadinene (9.3%), *E*-caryophyllene (8.3%), bicyclogermacrene (5.9%), α-cadinol (5.5%)	—	[25]
*E. protenta*	Santarém Novo, Pará, Brazil	Leaf (HD)	dimethylxanthoxylin (73.2%), limonene (5.9%)	—	[23]
*E. protenta*	Santarém Novo, Pará, Brazil	Leaf (HD)	dimethylxanthoxylin (83.0%)	—	[23]
*E. protenta*	Santarém Novo, Pará, Brazil	Leaf (HD)	selin-11-en-4α-ol (18.3%), β-elemene (16.9%), β-selinene (5.6%), δ-cadinene (5.5%), γ-selinene (5.1%)	—	[23]
*E. protenta*	Magalhães Barata, Pará, Brazil	Leaf (HD)	selin-11-en-4α-ol (14.4%), β-elemene (12.3%), α-selinene (6.5%), cubenol (5.7%)	—	[23]
*E. protenta*	Maracanã, Pará, Brazil	Leaf (HD)	β-elemene (9.2%), germacrene D (7.8%), α-cadinol (8.1%)	—	[23]
*E. protenta*	Maracanã, Pará, Brazil	Leaf (HD)	germacrene D (15.1%), bicyclogermacrene (11.8%), δ-cadinene (5.8%), α-copaene (5.1%), *E*-caryophyllene (5.1%)	—	[23]
*E. protenta*	Maracanã, Pará, Brazil	Leaf (HD)	δ-cadinene (15.4%), germacrene D (11.5%), δ-elemene (8.5%), *E*-caryophyllene (7.9%), α-copaene (6.2%)	—	[23]
*E. protenta*	Algodoal island, Maracanã, Pará, Brazil	Leaf (HD)	germacrene D (15.6%), δ-cadinene (8.5%), α-cadinol (6.9%), bicyclogermacrene (6.8%), *τ*-muurolol (5.9%), *E*-caryophyllene (5.7%)	—	[23]
*E. punicifolia*	Serra Negra, Pernambuco, Brazil	Leaf (HD)	linalool (44.0%), *E*-caryophyllene (22.7%), α-terpineol (8.8%)	—	[43]
*E. punicifolia*	Brejo da Madre de Deus, Pernambuco, Brazil	Leaf (HD)	linalool (61.2%), *E*-caryophyllene (16.2%), α-terpineol (6.7%)	—	[43]
*E. punicifolia*	Maracanã, Pará, Brazil	Leaf (HD)	*E*-caryophyllene (9.9%), bicyclogermacrene (8.7%), *E*-β-ocimene (5.5%), germacrene D (5.4%)	—	[25]
*E. punicifolia*	Macaé, Rio de Janeiro, Brazil	Leaf (HD)	α-cadinol (10.6%), 10-*epi*-γ-eudesmol (10.2%), paradisiol (9;0%), 7-*epi*-α-selinene (6.8%)	—	[28]
*E. pyriformis*	Porto Alegre, Rio Grande do Sul, Brazil	Leaf (HD)	α-cadinol (14.0%), δ-cadinene (12.4%), τ-cadinol (11.9%), bicyclogermacrene (10.2%)	—	[30]
*E. pyriformis*	Curitiba, Paraná, Brazil	Leaf (HD) ^b^	β-pinene (0.0-25.7%), limonene (0.2-22.0%), caryophyllene oxide (3.9-21.3%), 1,8-cineole (0.6-14.7%)	—	[44]
*E. pyriformis*	Curitiba, Paraná, Brazil	Flower (HD)	*E*-caryophyllene (22.8%), germacrene D (15.3%), bicyclogermacrene (8.4%), α-cadinol (7.2%), spathulenol (6.8%)	—	[44]
*E. pyriformis*	Curitiba, Paraná, Brazil	Fruits (HD)	caryophyllene oxide (16.2%), limonene (12.4%), α-terpineol (5.4%), α-cadinol (5.4%)	—	[44]
*E. ramboi*	Torres, Rio Gande do Sul, Brazil	Leaf (HD)	β-elemene (10.6%), bicyclogermacrene (9.7%), *E*-caryophyllene (6.2%)	—	[27]
*E. repanda*	Porto Rico, Paraná, Brazil	Leaf (HD)	*E*-caryophyllene (16.3%), α-humulene (10.2%), bicyclogermacrene (9.4%), germacrene D (7.5%), β-elemene (7.3%)	—	[61]
*E. selloi*	Rio de Janeiro, Rio de Janeiro, Brazil	Leaf (HD) ^b^	bicyclogermacrene (15.2-24.3%), germacrene D (5.7-18.7%), *E*-caryophyllene (6.7-12.5%), viridiflorol (3.5-9.1%)	—	[26]
*E. speciosa*	Itapuã, Rio Grande do Sul, Brazil	Leaf (HD)	α-pinene (47.3%), limonene (23.0%), γ-terpinene (2.5%)	—	[32]
*E. stictopetala*	São Luis, Maranhão, Brazil	Leaf (HD)	𝛾-elemene (17.48%), *E*-caryophyllene (16.46%), bicyclogermacrene (8.11%), 𝛽-pinene (7.08%), germacrene D (5.64%)	Larvicidal (*Aedes aegypti*, LC_50_ 230 μg/mL)	[63]
*E. stigmatosa*	Peruibe, São Paulo, Brazil	Leaf (HD)	physeteric acid (90.5%)	—	[62]
*E. stipitata*	São Miguel island, Açores, Portugal	Leaf (HD)	*E*-caryophyllene (22.7%), caryophyllene oxide (15.4%), α-pinene (14.1%), 4,8-α-epoxy-caryophyllene (10.9%) ^a^	Antibacterial, agar diffusion assay (*Staphylococcus aureus*, 14 mm; *Pseudomonas aeruginosa*, 11 mm*; Listeria monocytogenes*, 12 mm)	[77]
*E. stipitata*	Recife, Pernambuco, Brazil	Fruit (SPE)	germacrene D (38.3%), β-pinene (15.2%), α-pinene (10.4%), NI (12.6%)	—	[90]
*E. stipitata*	Florencia-Caquetá, Colombia	Fruit (SD)	limonene (81.0%), myrcene (5.5%)	—	[91]
*E. sulcata*	Restinga de Jurubatiba, Rio de Janeiro, Brazil	Leaf (HD)	*E*-caryophyllene (24.6%), α-pinene (17.2%), β-pinene (10.9%), 1,8-cineole (5.6%), α-humulene (5.1%)	Enzyme inhibitory (acetylcholinesterase, IC_50_ 4.66 μg/mL); Insecticidal (*Dysdercus peruvianus* and *Oncopeltus fasciatus*)	[45,46]
*E. sulcata*	Jureia, São Paulo, Brazil	Leaf (HD)	α-pinene (34.2%), *E*-caryophyllene (15.0%), globulol (11.8%), spathulenol (7.0%)	—	[32]
*E. sulcata*	Restinga de Jurubatiba, Rio de Janeiro, Brazil	Fine stem (HD)	*E*-caryophyllene (18.8%), spathulenol (8.8%), calamenene (7.0%), γ-cadinene (5.8%), α-humulene (5.6%), caryophyllene oxide (5.6%)	—	[46]
*E. sulcata*	Macaé, Rio de Janeiro, Brazil	Leaf (HD)	1,8-cineole (19.0%), α-pinene (16.9%), β-pinene (14.5%), caryophyllene oxide (9.8%)	—	[28]
*E. triquetra*	Pueblo Hondo, Táchira, Venezuela	Leaf (HD)	linalool (17.5%), limonene (16.9%), α-pinene (11.6%), β-pinene (8.7%)	Larvicidal (*Aedes aegypti*, LC_50_ 64.8 ppm)	[65]
*E. uruguayensis*	Torres, Rio Grande do Sul, Brazil	Leaf (HD)	α-pinene (23.5%), β-pinene (11.8%), *E*-caryophyllene (9.5%), caryophyllene oxide (6.4%)	—	[27]
*E. uniflora*	Pelotas, Rio Grande do Sul, Brazil	Leaf (HD)	germacrene B (21.2%), selin-1,3,7-(11)-trien-8-one epoxide (19.3%), *E*-caryophyllene (12.6%), germacrene A (11.6%), germacrene D (11.4%), selina-1,3,7-(11)-trien-8-one (9.7%)	Antimicrobial, agar diffusion assay and broth dilution assay (*Staphylococcus aureus*, MIC 800 μg/mL; *Listeria monocytogenes*, MIC 1040 μg/mL; *Candida lipolytica*, MIC 93.7 μg/mL; *C. guilliermondii*, MIC 109.4 μg/mL). Antioxidant (DPPH radical scavenging assay, IC_50_ 833 μg/mL; ABTS IC_50_ 8.1 μg/mL). Hepatoprotective effect in rats	[57,88]
*E. uniflora*	São Lourenço do Sul, Rio Grande do Sul, Brazil	Leaf (HD)	NI (38.28%), *E*-caryophyllene (8.18%), spathulenol (7.71%), 9,10-dehydro-*iso*-longifolene (6.21%), viridiflorol (5.78%), *allo*-aromadendrene (5.66%), dihydro-*cis*-α-copaene-8-ol (5.24%), δ-cadinene (5.19%), τ-cadinol (5.08%)	Antibacterial, broth dilution assay (*Enterobacter aerogenes* MIC 3.125%; *Salmonella typhimurium* (MIC 3.125%)	[78]
*E. uniflora*	São Luís, Maranhão, Brazil	Leaf (HD)	curzerene (47.3%), γ-elemene (14.2%), *trans*-β-elemenone (10.4%), β-elemene (5.5%)	Antileishmanial (*L. amazonensis* promastigotes, IC_50_ 3.04 μg/mL; *L. amazonensis* amastigotas, IC_50_ 1.92 μg/mL)	[47]
*E. uniflora*	Osasco, São Paulo, Brazil	Leaf (SD)	atractilone (26.78%), curzerene (17.96%), germacrene B (9.31%)	Antimicrobial, agar diffusion assay and broth dilution assay (*Streptococcus equi*, MIC 7500 μg/mL; *Staphylococcus epidermis*, MIC 7500 μg/mL; *Candida dubliniensis*, MIC 230 μg/mL;*C. tropicalis*, MIC 900 μg/mL; *C. albicans*, MIC 1800 μg/mL; *C. glabrata*, MIC 930 μg/mL; *C. parapsilosis*, MIC 3750 μg/mL; *C. grubii* serotype A, MIC 450 μg/mL; *C. gattii* serotype C, MIC 1800 μg/mL; *C. gattii* serotype B, MIC 220 μg/mL; *C. neoformans* serotype D, MIC 110 μg/mL; *Saccharomyces cerevisiae*, MIC 220 μg/mL)	[51]
*E. uniflora*	Águas de Santa Bárbara, São Paulo, Brazil	Leaf (SD)	selina-1,3,7-(11)-trien-8-one (34.0%), selina-1,3,7-(11)-trien-8-one epoxide (17.0%), germacrene B (10.5%), curzerene (5.5%), *E*-caryophyllene (5.0%)	—	[53]
*E. uniflora*	Botucatu, São Paulo, Brazil	Leaf (HD)	selina-1,3,7-(11)-trien-8-one (30.1%), selina-1,3,7-(11)-trien-8-one epoxide (21.89%), *E*-caryophyllene (6.1%)	Antibacterial, broth dilution assay (*Staphylococcus aureus*, MIC 55,600.0 μg/mL; *Staphylococcus aureus* – methicillin resistant, MIC 56,000.0 μg/mL; *Staphylococcus aureus* – methicillin sensitive, MIC 50,800.0 μg/mL; *Salmonella* spp., MIC 92,400.0 μg/mL; *Salmonella enteritidis*, MIC 92,400.0 μg/mL; *Salmonella typhimurium*, MIC 92,400.0 μg/mL; *Escherichia coli*, MIC 84,300.0 μg/mL; *Pseudomonas aeruginosa*, MIC 92,400.0 μg/mL)	[76]
*E. uniflora*	Seropédica, Rio de Janeiro, Brazil	Young leaf (HD)	germacrone (37.86%), curzerene (16.6%), germacrene B (13.59%), *E*-caryophyllene (6.02%)	—	[92]
*E. uniflora*	Seropédica, Rio de Janeiro, Brazil	Old leaf (HD)	curzerene (22.37%), furanodiene (18.99%), germacrene B (14.39%), *E*-caryophyllene (9.35%)	—	[92]
*E. uniflora*	Anápolis, Goiás, Brazil	Aerial parts (HD) ^b^	selina-1,3,7-(11)-trien-8-one (43.0%), selina-1,3,7-(11)-trien-8-one epoxide (20.0-29.0%), spathulenol (7.5-10.0%)	—	[54]
*E. uniflora*	Belém, Pará, Brazil	Aerial parts (HD)	germacrone (32.8%), curzerene (30.0%), germacrene B (15.6%)	—	[55]
*E. uniflora*	Santarém, Pará, Brazil	Leaf (HD)	selina-1,3,7(11)-trien-8-one (32.6%), selina-1,3,7(11)-trien-8-one epoxide (30.4%), *E*-caryophyllene (5.4%), germacrene B (5.0%)	Antioxidant, DPPH radical scavenging assay (30.3%, 153.5 mg.TE/mL); Antioxidant, β-carotene/linoleic acid assay at 1 mg/mL, (10.8%)	[48]
*E. uniflora*	Belém, Pará, Brazil	Leaf (HD)	selina-1,3,7(11)-trien-8-one (43.1%), selina-1,3,7(11)-trien-8-one epoxide (21.7%), germacrene B (5.9%)	Antioxidant, DPPH radical scavenging assay (35.3%, 178.8 mg.ET/mL); Antioxidant, β-carotene/linoleic acid assay at 1 mg/mL (23.0%); Cytotoxic (HCT- human colon carcinoma, IC_50_ 16.26 μg/mL; MRC5 human fibroblast IC_50_ 10.27 μg/mL; AGP-01 human gastric adenocarcinoma, IC_50_ 12.60 μg/mL; SKMEL-19 human melanoma, IC_50_ 12.20 μg/mL)	[48]
*E. uniflora*	Belém, Pará, Brazil	Leaf (HD)	caryophyllene oxide (18.1%), selina-1,3,7(11)-trien-8-one (18.1%), selina-1,3,7(11)-trien-8-one epoxide (16.0%), β-elemene (8.9%), *E*-caryophyllene (6.1%)	Antioxidant, DPPH assay (45.1%, 228.3 mg.ET/mL); Antioxidant, β-carotene/linoleic acid assay at 1mg/mL (16.8%); Cytotoxic (HCT-116 human colon carcinoma, IC_50_ > 25 μg/mL; MRC5 human fibroblast IC_50_ > 25 μg/mL; AGP-01 human gastric adenocarcinoma, IC_50_ > 25 μg/mL; SKMEL-19 human melanoma, IC_50_ >25 μg/mL)	[48]
*E. uniflora*	Bahia, Brazil	Leaf (SPME)	germacrene B (9.5%), γ-elemene (8.5%), β-elemene (7.4%), *E*-caryophyllene (7.1%), germacrene D (5.5%), γ-muurolene (5.3%), germacrone (5.1%)	—	[58]
*E. uniflora*	Belém, Pará, Brazil	Leaf (HD)	curzerene (50.6%), germacrene B (5.3%)	Antioxidant, DPPH assay (42.8%, 217.0 mg.ET/mL); Antioxidant, β-carotene/linoleic acid assay at 1mg/mL (20.1%) Cytotoxic (HCT-116 human colon carcinoma, IC_50_ 9.28 μg/mL; MRC5 human fibroblast IC_50_ 14.95 μg/mL; AGP-01 human gastric adenocarcinoma, IC_50_ 8.73 μg/mL; SKMEL-19 human melanoma, IC_50_ 15.42 μg/mL)	[48]
*E. uniflora*	Belém, Pará, Brazil	Leaf (HD) ^b^	curzerene (34.4-53.1%), germacrone (0.2–10.5%), globulol (1.5–7.4%), germacrene B (0.1–7.5%), spathulenol (0.5–7.0%), viridiflorol (0.8–6.2%), β-elemene (1.8–5.8%)	Antioxidant, DPPH assay (42.6-64.2%, 186.9-400.3 mg.ET/g)	[73]
*E. uniflora*	Belém, Pará, Brazil	Leaf (HD)	germacrene B (18.4%), curzerene (13.4%), *E*-caryophyllene (9.1%), γ-elemene (7.8%), β-elemene (7.4%)	Antioxidant, DPPH assay (40.6%, 205.6 mg.ET/mL); Antioxidant β-carotene/linoleic acid (26.3%); Cytotoxic (HCT-116 human colon carcinoma, IC_50_ > 25 μg/mL; MRC5 human fibroblast IC50 > 25 μg/mL; AGP-01 human gastric adenocarcinoma, IC_50_ > 25 μg/ML; SKMEL-19 human melanoma, IC_50_ >25 μg/mL)	[48]
*E. uniflora*	Bahia, Brazil	Leaf (SPME)	germacrene B (9.0%), γ-elemene (8.1%), γ-muurolene (7.7%), germacrene D (6.1%), β-elemene (5.0%)	—	[58]
*E. uniflora*	Bahia, Brazil	Leaf (SPME)	germacrene B (9.6%), β-elemene (9.3%), γ-elemene (8.0%), germacrene D (6.5%), *E*-caryophyllene (5.2%), γ-muurolene (5.0%)	—	[58]
*E. uniflora*	Rio de Janeiro, Rio de Janeiro, Brazil	Leaf (HD)	atractilone, 3-furanoeudesmene	Antinociceptive (ED_50_ 218.6 mg/kg) and hypothermic in mouse model	[52]
*E. uniflora*	Rio de Janeiro, Rio de Janeiro, Brazil	Leaf (HD)	curzerene (50.2%), β-elemene (5.9%)	—	[50]
*E. uniflora*	Goiás, Brazil	Leaf (HD)	germacrene B (21.6%), curzerene (20.5%), germacrone (17.3%), *E*-caryophyllene (8.7%)	Antifungal, broth dilution assay (*Paracoccidioides brasiliensis*, MIC 500 µg/mL; MFC 500 µg/mL)	[49]
*E. uniflora*	Goiás, Brazil	Leaf (HD)	curzerene (42.6%), germacrone (13.5%), germacrene D (8.8%), germacrene A (7.4%), *E*-caryophyllene (7.0%)	Antifungal, broth dilution assay (*Paracoccidioides brasiliensis*, MIC 62.5 µg/mL; MFC 125 µg/mL)	[49]
*E. uniflora*	Goiás, Brazil	Leaf (HD)	selin-1,3,7-(11)-trien-8-one (48.2%), selin-1,3,7-(11)-trien-8-one epoxide (19.3%), atractilone (11.7%), germacrene B (5.9%)	Antifungal, broth dilution assay (*Paracoccidioides brasiliensis*, MIC 250 µg/mL; MFC 250 µg/mL)	[49]
*E. uniflora*	Pelotas, Rio Grande do Sul, Brazil	Fruits (HD)	hexadecanoic acid (11.7%)*, E*-β-ocimene (7.4%), α-selinene (7.2%), germacrene B (7.2%), bicyclogermacrene (5.2%)	Antioxidant, DPPH assay (TLC method)	[40]
*E. verticillata*	Caraguatatuba, São Paulo, Brazil	Leaf (HD)	valerianol (28.1%), 10-*epi*-γ-eudesmol (12.6%), *E*-caryophyllene (10.9%), α-selinene (6.1%), δ-cadinene (5.7%)	Enzyme inhibitory (acetylcholinesterase, IC_50_ 67.3 μg/mL)	[64]
*E. xiriricana*	Peruíbe, São Paulo, Brazil	Leaf (HD)	spathulenol (15.4%), β-pinene (14.1%), globulol (8.6%), α-pinene (7.8%)	—	[32]
*S. cumini*	Rio de Janeiro, Rio de Janeiro, Brazil	Leaf (HD)	α-pinene (22.2%), *Z*-β-ocimene (10.2%), *E*-caryophyllene (9.45%), limonene (7.3%), α-terpineol (7.00%), *E*-β-ocimene (5.88%), α-humulene (5.5%)	Anti-inflammatory, lipopolysaccharide-induced pleurisy model, an eosinophil was inhibited in 67%	[66]
*S. cumini*	São Luis, Maranhão, Brazil	Leaf (HD)	α-pinene (31.85%), *Z*-β-ocimene (28.98%), *E*-β-ocimene (11.71%), β-pinene (5.57%), *E*-caryophyllene (5.02%)	Antileishmanial (*Leishmania amazonensis* promastigotes, IC_50_ 60 μg/mL); Molluscicide (*Biomphalaria glabrata*, LC_50_ 90 μg/mL)	[67]
*S. cumini*	Crato, Ceará, Brazil	Leaf (HD)	α-pinene (48.1%), *E*-nerolidol (8.7%), nerol (7.1%), nonanol (6.8%) ^a^	Antibacterial, broth dilution assay (*Staphylococcus aureus*, MIC 128 µg/mL; *Escherichia coli* MIC > 1024 μg/mL)	[68]
*S. cumini*	Crato, Ceará, Brazil	Leaf (HD)	α-pinene (30.0%), *E*-β-ocimene (26.8%), β-ocimene (11.1%), β-pinene (8.3%), l imonene (6.5%), β-Fenchol (7.3%) ^a^	—	[69]
*S. cumini*	Juiz de Fora, Minas Gerais, Brazil	Leaf (HD)	α-humulene (25.24%), *E*-caryophyllene (16.00%), α-terpineol (9.08%), globulol (5.23%)	Anti-inflammatory; Antibacterial (*Mycobacterium bovis*)	[70]
*S. cumini*	Porto Rico, Paraná, Brazil	Seed (HD) ^b^	*E*-caryophyllene (6.4-42.5%), α-humulene (6.6-22.2%), caryophyllene oxide (5.3-37.3%), humulene epoxide II (2.1-17.1%)	—	[93]
*S. jambos*	Rio Verde, Goiás, Brazil	Leaf (HD) ^b^	*E*-caryophyllene (0–10.86%), α-humulene (0.0-7.07%), α-zingiberene (0.97–17.73%), hydroxytoluenebuthyled (3.44–32.82%), caryophyllenyl alcohol (1.48–17.14%), caryolan-8-ol (0–10.75%), caryophyllene oxide (0–5.05%), tujopsan-2-α-ol (1.01–12.19%), heneicosane (1.73–18.0%) ^a^	—	[74]

^a^ = The analysis is doubtful, based on known retention indices (RI), ^b^ = seasonal study, SD = steam distillation, HD = hydrodistillation, SPME = solid phase microextraction, NI = unidentified compound, IC_50_ = median inhibitory concentration, MIC = minimum inhibitory concentration, MMC= minimum microbicide concentration, LC_50_ = median lethal concentration.
